# Structural and temporal dynamics of nano-based therapies in ulcerative colitis: history, hotspots, and emerging trends

**DOI:** 10.3389/fimmu.2026.1739037

**Published:** 2026-02-18

**Authors:** Junzu Shi, Danyang Cui, Li Yu, Yang Gong

**Affiliations:** Department of Traditional Chinese Medicine, General Hospital of Northern Theater Command, Shenyang, Liaoning, China

**Keywords:** bibliometrics, emerging frontiers, nanotherapeutics, research trends, ulcerative colitis

## Abstract

**Background:**

Nanomaterial-based therapies have emerged as a promising approach for ulcerative colitis (UC). To systematically map the field, we performed a bibliometric analysis of research over the past 25 years, aiming to reveal knowledge evolution, core themes, and emerging trends.

**Methods:**

We searched the Web of Science Core Collection and Scopus for English-language publications (2001–2025) using the keywords “nanomaterials” and “ulcerative colitis” including original articles and reviews. After deduplication, 1,316 publications were analyzed using CiteSpace, HistCite, and R to construct collaboration networks, keyword co-occurrence maps, co-citation networks, and thematic evolution analyses.

**Results:**

UC-focused nanotherapeutics show sustained growth and increasing international collaboration, with China as the leading contributor. Analysis identified 57 subject categories, 537 keywords, and 899 publications with citation bursts. Five emerging themes were highlighted: gut microbiota, reactive oxygen species, NLRP3 inflammasome, drug delivery systems, and polymeric nanoparticles. Nanotechnology is increasingly integrated into UC pathogenesis studies, with a shift from passive drug delivery toward intelligent, mechanism-driven, and combination therapies. Co-citation analysis identified seven core areas, including inflammation regulation, targeted delivery, immune modulation, hydrogel carriers, and nanotherapeutics.

**Conclusion:**

This study maps the evolving landscape of UC nanotherapeutics, highlighting the field’s shift toward precise, intelligent, and mechanism-guided strategies. Future research should emphasize nanodesign, safety evaluation, and personalized therapies, facilitating translational innovation.

## Graphical Abstract

**Figure:**
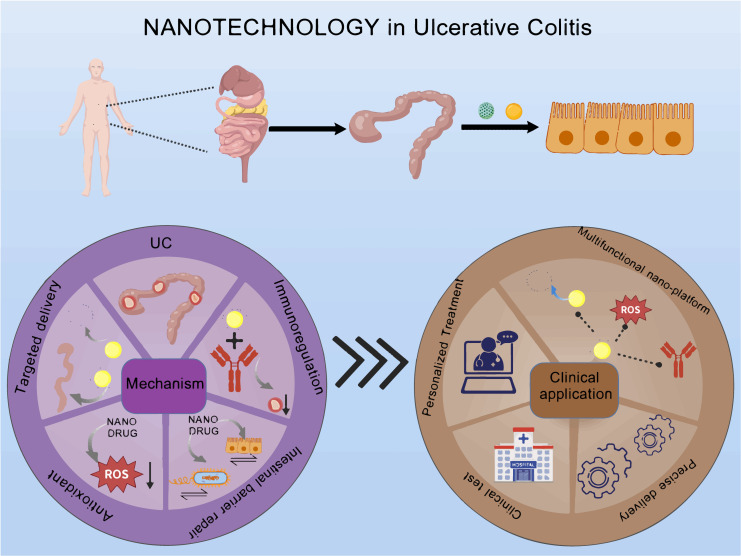

## 1 Introduction

Ulcerative colitis (UC) is a chronic, non-specific inflammatory disorder primarily affecting the rectum and colon, clinically characterized by abdominal pain, diarrhea, and mucous-purulent stools (1, 2). Although its precise pathogenesis remains unclear, UC is closely associated with environmental factors, genetic predispositions, immune dysregulation, and microbiota imbalance (3). Therapeutic strategies aim to achieve clinical remission and improve patients’ quality of life (4). Conventional treatments, including aminosalicylates, immunosuppressants, corticosteroids, and biologics, are limited by potential drug resistance and adverse effects (5). Consequently, safer and more effective therapeutic alternatives are urgently needed. Consequently, safer and more effective therapeutic alternatives are urgently needed.

Nanotechnology-based therapies have emerged as a promising approach, offering the potential to improve drug delivery and therapeutic efficacy in UC (6, 7). By enabling targeted and controlled delivery, these strategies can enhance local drug accumulation at lesion sites while reducing systemic exposure (8, 9). This rationale underpins growing interest in nanotherapeutics for UC and motivates a systematic evaluation of research trends in this field.

Bibliometrics provides a quantitative and qualitative framework to assess scholarly literature, allowing rapid mapping of research structures, collaboration networks, knowledge evolution, and emerging hotspots (10, 11). Despite increasing research on nano-based therapies for UC, no comprehensive bibliometric analysis has yet been conducted. This study presents a bibliometric and visualization analysis of literature on nanotechnology applications in UC, encompassing authors, institutions, countries, journals, co-cited references, and keywords. The aim is to elucidate the intellectual structure, highlight research hotspots, and provide a data-driven foundation to guide future mechanistic and translational studies.

## 2 Materials and methods

### 2.1 Data source and search strategy

We conducted a bibliometric analysis using publications retrieved from two major academic databases: the Web of Science Core Collection (WoSCC) and Scopus, selected for their extensive coverage of peer-reviewed literature. The search was restricted to English-language articles and reviews published between 2001 and 2025. The final retrieval was performed on July 1, 2025, ensuring a consistent cutoff for all included records. The full search strategy is provided in **Supplementary Material** (**Supplementary Table S1**). Initial retrieval yielded 961 records from WoSCC and 949 from Scopus.

To address overlap between databases, duplicate records were identified and removed using the R -bibliometrix package (v4.3.0), which matches entries based on title, author list, publication year, and DOI (12). After deduplication and application of eligibility criteria (i.e., inclusion of only original research articles and review papers), the final dataset was used for all subsequent analyses.

### 2.2 Bibliometric analysis methods

A multi-method bibliometric approach was employed. Co-occurrence networks of keywords, authors, institutions, and countries were constructed using CiteSpace (v6.2.R3) to visualize collaboration patterns and thematic structures. Publications were divided into four time slices (2001–2007, 2008–2013, 2014–2019, 2020–2025) for cluster analysis based on the Log-Likelihood Ratio (LLR) algorithm. Kleinberg’s burst detection algorithm identified emerging topics, while betweenness centrality highlighted pivotal nodes in the networks.

**Figure 1:**
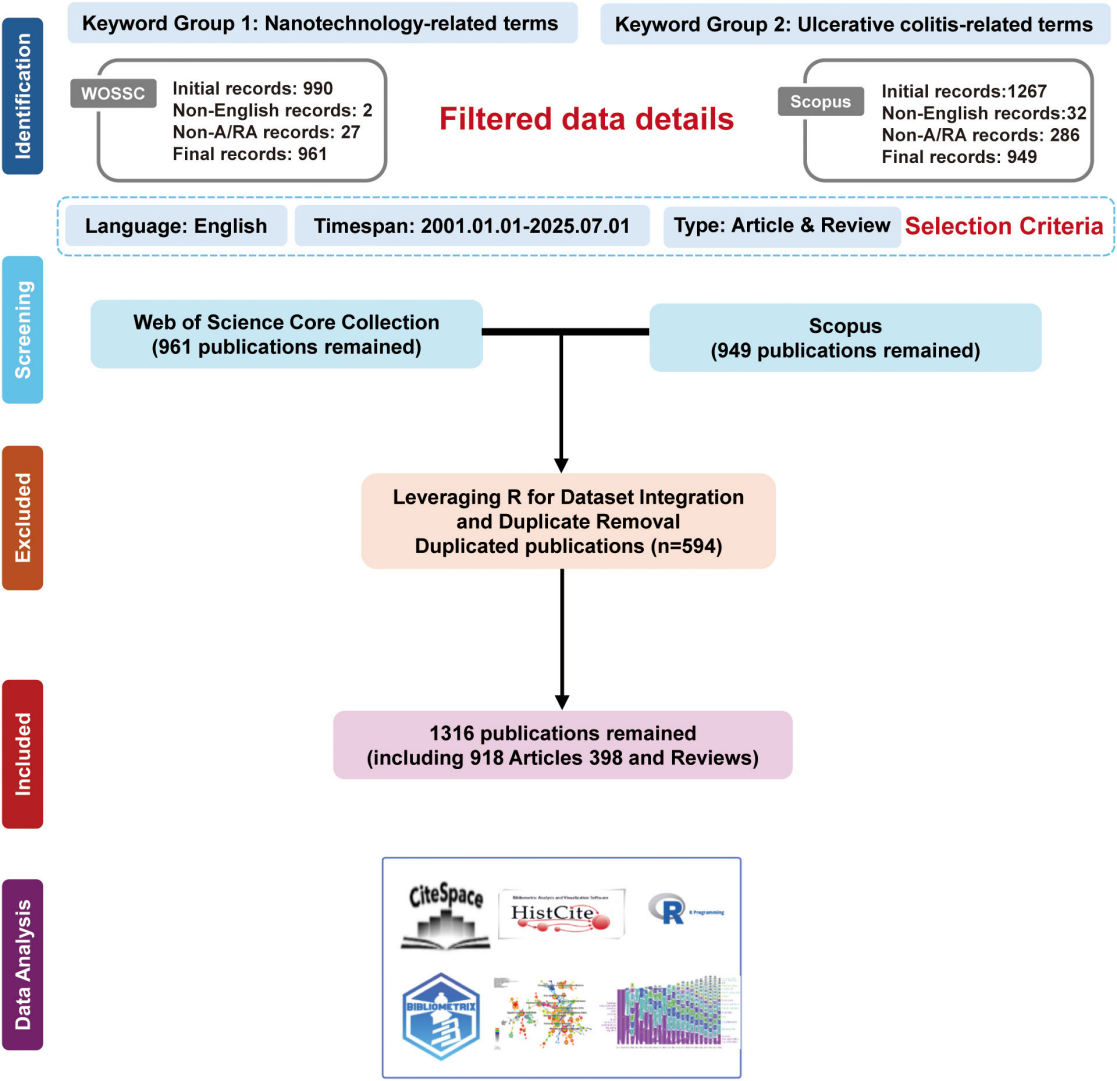
The flowchart of the literature screening process in this study. Depicts the workflow for literature screening and dataset integration. A total of 990 records were retrieved from WOSCC and 1,267 from Scopus. After excluding non-English and non-article/review records and applying filters for document type and publication period (January 2001–July 2025), 961 WOSCC and 949 Scopus publications were retained. The higher number of non-A/RA records in Scopus reflects its broader indexing policy. Duplicate removal using R eliminated 594 overlapping records, yielding 1,316 unique publications (918 articles and 398 reviews) for analysis. Bibliometric analyses were subsequently conducted using CiteSpace, HistCite, and R to map research trends, collaboration networks, and thematic evolution.

Intellectual development was traced through reference-based clustering and citation timeline graphs, supplemented by burstiness maps for keywords and references. HistCite Pro (v2.1) was used to compute Local and Global Citation Scores (LCS/GCS) to identify seminal works. An Alluvial Flow Diagram was generated from CiteSpace keyword co-occurrence data to illustrate thematic evolution. Trend analysis was performed in R, with donut charts depicting the temporal distribution of research categories (**Figure 1**). All raw data were preprocessed in Microsoft Excel (WPS 2021) for cleaning and descriptive summarization prior to specialized bibliometric analysis.

## 3 Results

### 3.1 The historical features of the literature on nano-based therapies in UC

#### 3.1.1 Distribution of publications

**Figure 2:**
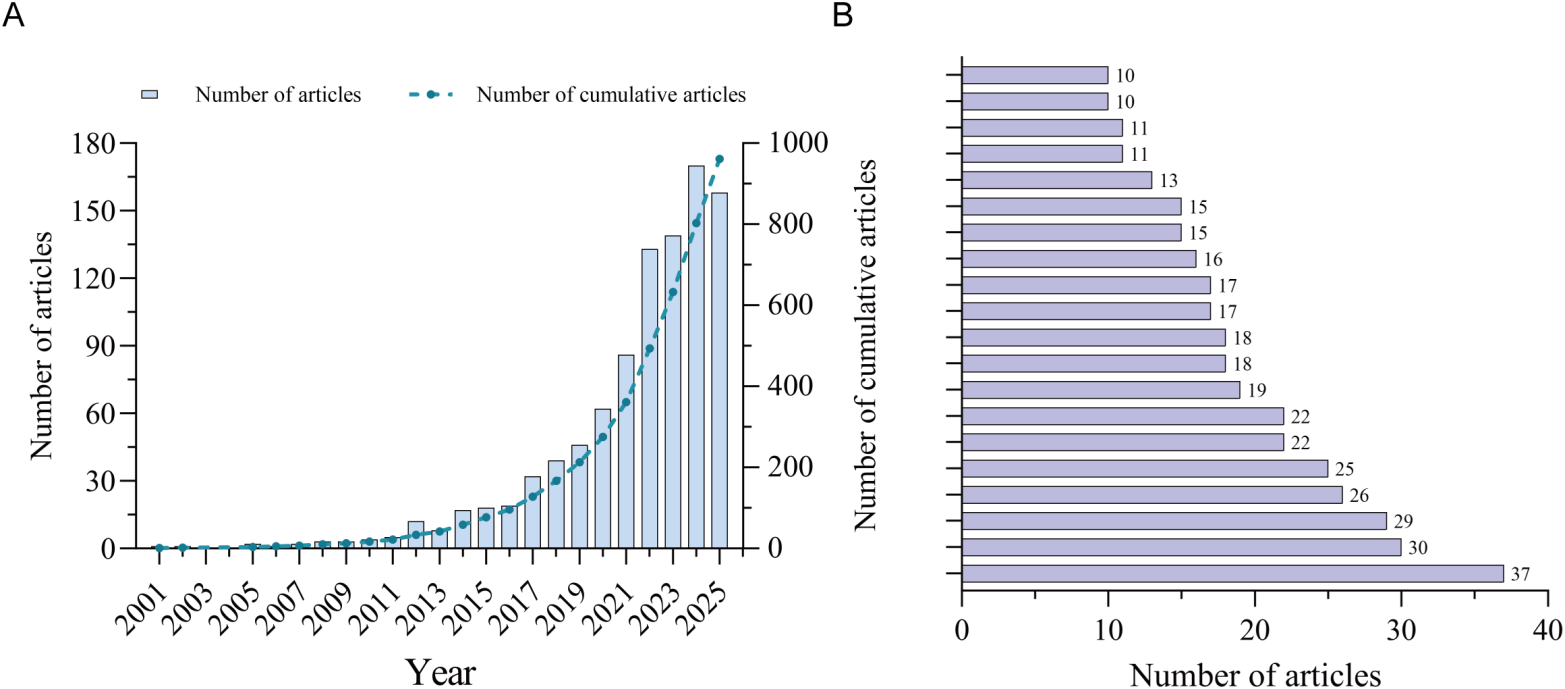
**(A)** Exponential growth in UC nanotherapy publications since 2011, with a sharp rise after 2020 to over 140 articles in 2025. **(B)** Highlights leading journals—International Journal of Biological Macromolecules, Journal of Controlled Release, and International Journal of Nanomedicine—reflecting a focus on biomaterials, drug delivery, and translation. The absence of clinical journals suggests the field remains rooted in materials science and biomedical engineering, marking its evolution from a niche topic to a robust interdisciplinary research area.

A total of 961 publications on nanomaterials in UC were retrieved from the Web of Science Core Collection (WOSCC), including 751 research articles and 210 reviews, authored by 4,900 researchers from 1,299 institutions, published in 317 journals across 62 subject categories (**Table 1**). Annual publication output remained below five per year from 2001 to 2013, increased steadily between 2014 and 2020, and surged after 2021, peaking in 2024 (**Figure 2A**). The top three journals by publication count were International Journal of Biological Macromolecules (37), Journal of Controlled Release (30), and International Journal of Nanomedicine (29); the top 20 are listed in **Figure 2B**.

**Table 1:** Quantity in publication output for nano-based therapies applications in UC from 2001 to 2025.

Categories	Publication	Articles	Review	Authors	Institutions	Journals	Subject categories
Amount	961	751	210	4900	1299	317	62

#### 3.1.2 The research trajectory of nano-based therapies in UC

**Figure 3:**
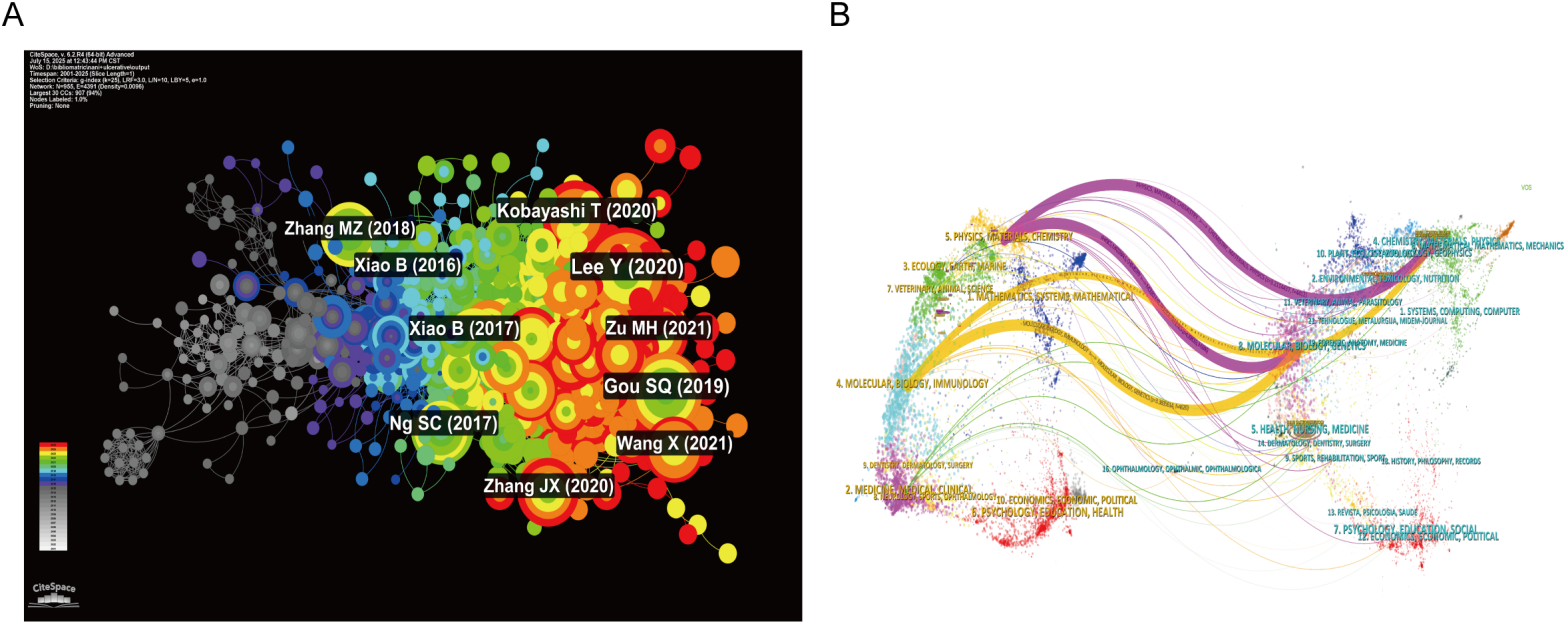
Journals visualization analysis. **(A)** Co-citation network of references **(B)** Dual-map overlay of journals,illustrating cross-disciplinary citation flows. The orange path indicates that research published in journals categorized under Molecular Biology/Immunology is predominantly cited by journals in Molecular Biology/Genetics. The pink path shows that work originating from Physics/Materials/Chemistry journals is mainly cited by literature in Molecular Biology/Genetics, highlighting knowledge transfer from materials science into biological domains.

The co-citation network comprised 955 nodes and 4,391 links (**Figure 3A**). Ten publications received the highest co-citation frequencies: Lee Y (2020, 90), Gou SQ (2019, 64), Kobayashi T (2020, 57), Xiao B (2017, 53), Ng SC (2017, 52), Zhang JX (2020, 50), Xiao B (2016, 48), Zu MH (2021, 47), Wang X (2021, 45), and Le Berre C (2023, 45). The dual-map overlay identified two main citation paths: one from Molecular Biology/Immunology to Molecular Biology/Genetics (orange path), and another from Physics/Materials/Chemistry to Molecular Biology/Genetics (pink path) (**Figure 3B**). The citation timeline graph and HisCite Pro 2.1 visualization identified three seminal references with the largest node sizes (**Table 2**).

**Table 2:** Information on the top 30 literature scored by LCS score.

NO.	Article information	Journal	LCS	GCS
1	Size-dependent bioadhesion of micro- and nanoparticulate carriers to the inflamed colonic mucosa	PHARM RES-DORDR	98	442
81	Combination Therapy for Ulcerative Colitis: Orally Targeted Nanoparticles Prevent Mucosal Damage and Relieve Inflammation	THERANOSTICS	94	190
221	Hyaluronic acid-bilirubin nanomedicine for targeted modulation of dysregulated intestinal barrier, microbiome and immune responses in colitis	NAT MATER	92	526
68	Advances in oral nano-delivery systems for colon targeted drug delivery in inflammatory bowel disease: Selective targeting to diseased versus healthy tissue	NANOMED-NANOTECHNOL	90	417
17	Orally delivered thioketal nanoparticles loaded with TNF-α-siRNA target inflammation and inhibit gene expression in the intestines	NAT MATER	81	597
91	Edible ginger-derived nanoparticles: A novel therapeutic approach for the prevention and treatment of inflammatory bowel disease and colitis-associated cancer	BIOMATERIALS	79	639
115	Orally Targeted Delivery of Tripeptide KPV via Hyaluronic Acid-Functionalized Nanoparticles Efficiently Alleviates Ulcerative Colitis	MOL THER	74	150
200	Multi-bioresponsive silk fibroin-based nanoparticles with on-demand cytoplasmic drug release capacity for CD44-targeted alleviation of ulcerative colitis	BIOMATERIALS	71	199
30	Nano- and microparticulate drug carriers for targeting of the inflamed intestinal mucosa	J CONTROL RELEASE	69	201
137	Oral Delivery of Nanoparticles Loaded With Ginger Active Compound, 6-Shogaol, Attenuates Ulcerative Colitis and Promotes Wound Healing in a Murine Model of Ulcerative Colitis	J CROHNS COLITIS	64	174
14	Drug-Loaded Nanoparticles Targeted to the Colon With Polysaccharide Hydrogel Reduce Colitis in a Mouse Model	GASTROENTEROLOGY	63	203
148	Nanoparticle-Based Oral Drug Delivery Systems Targeting the Colon for Treatment of Ulcerative Colitis	INFLAMM BOWEL DIS	62	141
95	A superoxide dismutase/catalase mimetic nanomedicine for targeted therapy of inflammatory bowel disease	BIOMATERIALS	56	195
237	Macrophage-based nanotherapeutic strategies in ulcerative colitis	J CONTROL RELEASE	51	222
159	TNFα gene silencing mediated by orally targeted nanoparticles combined with interleukin-22 for synergistic combination therapy of ulcerative colitis	J CONTROL RELEASE	51	109
48	Drug delivery strategies in the therapy of inflammatory bowel disease	ADV DRUG DELIVER REV	51	122
12	Transferrin as a Luminal Target for Negatively Charged Liposomes in the Inflamed Colonic Mucosa	MOL PHARMACEUT	50	135
33	An Orally Administered Redox Nanoparticle That Accumulates in the Colonic Mucosa and Reduces Colitis in Mice	GASTROENTEROLOGY	46	157
328	Oral Core-Shell Nanoparticles Embedded in Hydrogel Microspheres for the Efficient Site-Specific Delivery of Magnolol and Enhanced Antiulcerative Colitis Therapy	ACS APPL MATER INTER	45	159
173	Nanoparticle-Mediated Drug Delivery Systems For The Treatment Of IBD: Current Perspectives	INT J NANOMED	45	124
11	pH-Sensitive nanospheres for colon-specific drug delivery in experimentally induced colitis rat model	EUR J PHARM BIOPHARM	45	104
131	Colon-targeted delivery of cyclosporine A using dual-functional Eudragit^®^ FS30D/PLGA nanoparticles ameliorates murine experimental colitis	INT J NANOMED	43	87
36	Nano- and microscaled particles for drug targeting to inflamed intestinal mucosa-A first *in vivo* study in human patients	J CONTROL RELEASE	41	191
218	Amelioration of ulcerative colitis via inflammatory regulation by macrophage-biomimetic nanomedicine	THERANOSTICS	40	1232
28	Oral drug delivery with polymeric nanoparticles: The gastrointestinal mucus barriers	ADV DRUG DELIVER REV	40	115
261	Colitis-targeted hybrid nanoparticles-in-microparticles system for the treatment of ulcerative colitis	ACTA BIOMATER	39	61
252	Curcumin Nanocrystal/pH-Responsive Polyelectrolyte Multilayer Core-Shell Nanoparticles for Inflammation-Targeted Alleviation of Ulcerative Colitis	BIOMACROMOLECULES	36	218
117	Oral administration of ginger-derived nanolipids loaded with siRNA as a novel approach for efficient siRNA drug delivery to treat ulcerative colitis	NANOMEDICINE-UK	36	82
182	Advances in orally-delivered pH-sensitive nanocarrier systems; an optimistic approach for the treatment of inflammatory bowel disease	INT J PHARMACEUT	34	98
38	Galactosylated trimethyl chitosan-cysteine nanoparticles loaded with Map4k4 siRNA for targeting activated macrophages	BIOMATERIALS	33	141

#### 3.1.3 Scientific cooperation

**Figure 4:**
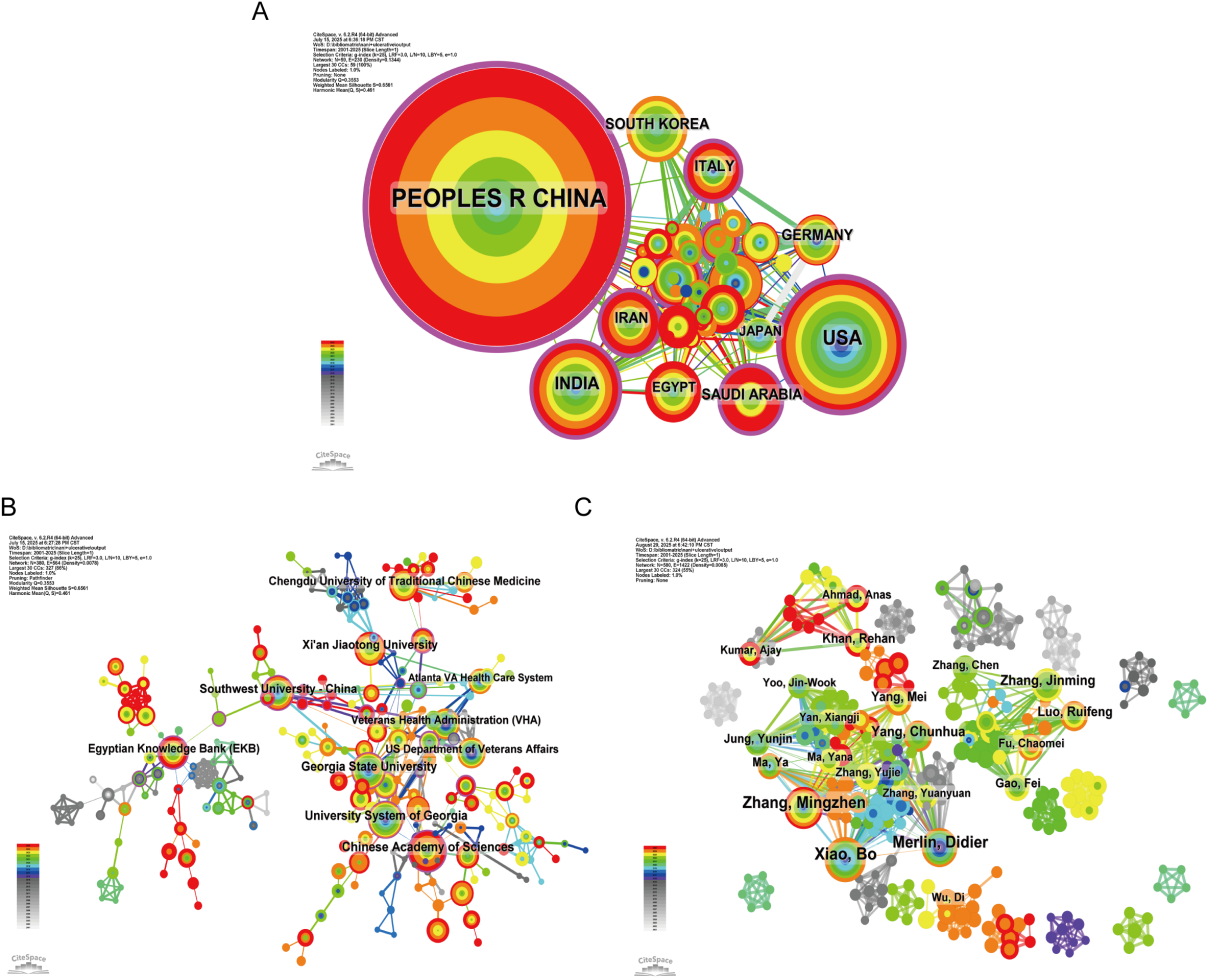
The scientific cooperation network. **(A)** Country cooperation includes 59 countries and 230 links, with China leading, followed by the U.S., India, Iran, and South Korea. While China forms a dense domestic cluster, multiple links to Western and Middle Eastern countries indicate growing international engagement, though global collaboration remains largely hub-and-spoke. **(B)** Institution cooperation highlights the Chinese Academy of Sciences, University System of Georgia, Georgia State University, and Egyptian Knowledge Bank (EKB). EKB’s high connectivity despite lower output suggests its role as a regional knowledge hub, while U.S. institutions reflect translational nanomedicine efforts. **(C)** The author network centers on Xiao Bo, Merlin Didier, Zhang Mingzhen, and Yang Chunhua, forming tightly connected subgroups that reveal distinct domestic and international research teams driving the field forward.

The country collaboration network included 59 countries and 230 links, with China, the United States, India, Iran, and South Korea as the most active (**Figure 4A**). The institutional network contained 380 nodes and 564 links; leading institutions were the Chinese Academy of Sciences, University System of Georgia, and Georgia State University (**Figure 4B**). The author collaboration network showed high connectivity among Xiao Bo, Merlin Didier, Zhang Mingzhen, and Yang Chunhua (**Figure 4C**). Full cooperation data are provided in **Supplementary Table S3**.

### 3.2 Variation of the most active topics

#### 3.2.1 Subject category burst

Of 62 subject categories, 57 exhibited citation bursts between 2001 and 2025. Gastroenterology & Hepatology had the highest burst intensity (8.79, 2007–2020). Subsequent bursts occurred in Pharmacology & Pharmacy (2012–2014), Biotechnology & Applied Microbiology (2016–2017), Nutrition & Dietetics (2018–2019), Toxicology (2020–2023), and Physics, Condensed Matter (2024–2025). Twenty new disciplines are projected to emerge post-2025, led by Physics, Condensed Matter, Food Science & Technology, and Chemistry, Applied (**Supplementary Figure S1**).

#### 3.2.2 Keywords burst

**Figure 5:**
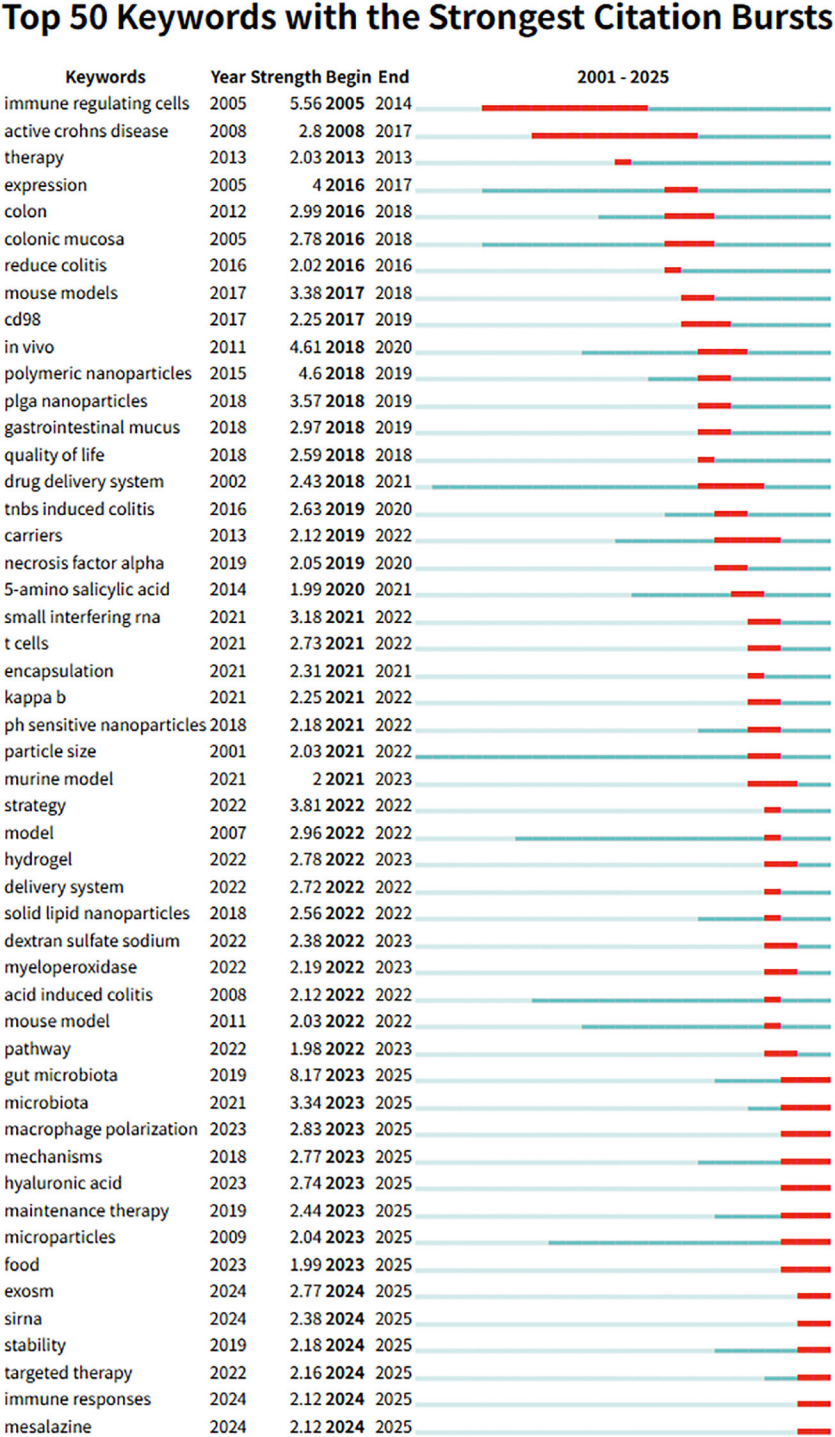
The top 50 keywords with the strongest citation bursts.

Among 537 burst keywords, the top 50 by intensity are shown in **Figure 5**, with full data in **Supplementary Table S4**. Gut microbiota exhibits the highest burst intensity (8.17, 2023–2025), alongside recent bursts for macrophage polarization and exosome (2023–2025). Earlier bursts involved immune-regulating cells (2005–2014) and polymeric nanoparticles (2018–2019). Citation burst analysis highlights the field’s evolution from foundational studies on immune mechanisms and stable carriers toward microbiome modulation, immune reprogramming, and extracellular vesicle signaling. Declining bursts for mouse models and drug delivery system suggest preclinical maturation, while emerging terms such as hyaluronic acid, siRNA, and targeted therapy reflect a shift toward smart, multi-targeted, and precision-oriented nanotherapies integrating immunomodulation, microbiome targeting, and personalized treatment.

#### 3.2.3 Reference burst

A total of 899 references exhibited citation bursts. The top three by burst strength were: Combination Therapy for Ulcerative Colitis: Orally Targeted Nanoparticles Prevent Mucosal Damage and Relieve Inflammation (2017–2021), Orally Targeted Delivery of Tripeptide KPV via Hyaluronic Acid-Functionalized Nanoparticles Efficiently Alleviates Ulcerative Colitis (2018–2022), and Advances in Oral Nano-delivery Systems for Colon-Targeted Drug Delivery in Inflammatory Bowel Disease (**Table 3**). From 2025 onward, 159 references are projected to burst; the top 20 are listed in **Table 4**.

**Table 3:** The references with citation bursts at different period.

References	Year	Strength	Begin	End	2001 - 20255
Wilson DS, 2010, NAT MATER, V9, P923, DOI 10.1038/nmat2859, DOI	2010	9.06	2012	2015	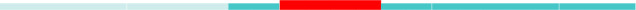
Laroui H, 2011, BIOMATERIALS, V32, P1218, DOI 10.1016/j.biomaterials.2010.09.062, DOI	2011	8.72	2012	2016	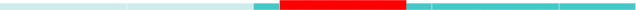
Laroui H, 2010, GASTROENTEROLOGY, V138, P843, DOI 10.1053/j.gastro.2009.11.003, DOI	2010	7.76	2012	2015	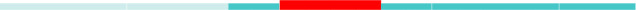
Xiao B, 2013, BIOMATERIALS, V34, P7471, DOI 10.1016/j.biomaterials.2013.06.008, DOI	2013	11.75	2014	2018	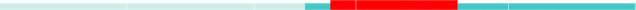
Schmidt C, 2013, J CONTROL RELEASE, V165, P139, DOI 10.1016/j.jconrel.2012.10.019, DOI	2013	11.75	2014	2018	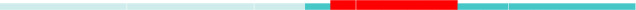
Coco R, 2013, INT J PHARMACEUT, V440, P3, DOI 10.1016/j.ijpharm.2012.07.017, DOI	2013	8.21	2014	2018	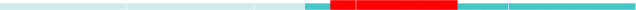
Collnot EM, 2012, J CONTROL RELEASE, V161, P235, DOI 10.1016/j.jconrel.2012.01.028, DOI	2012	8.12	2014	2017	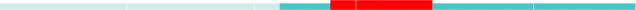
Beloqui A, 2014, INT J PHARMACEUT, V473, P203, DOI 10.1016/j.ijpharm.2014.07.009, DOI	2014	11.88	2015	2019	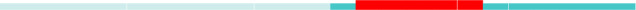
Xiao B, 2014, GASTROENTEROLOGY, V146, P1289, DOI 10.1053/j.gastro.2014.01.056, DOI	2014	11.31	2015	2019	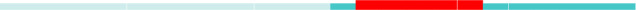
Laroui H, 2014, J CONTROL RELEASE, V186, P41, DOI 10.1016/j.jconrel.2014.04.046, DOI	2014	9.61	2015	2019	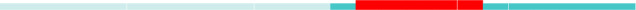
Ali H, 2014, J CONTROL RELEASE, V183, P167, DOI 10.1016/j.jconrel.2014.03.039, DOI	2014	9.04	2015	2019	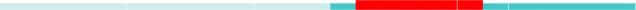
Hua S, 2015, NANOMED-NANOTECHNOL, V11, P1117, DOI 10.1016/j.nano.2015.02.018, DOI	2015	16.34	2016	2020	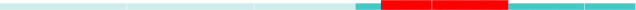
Zhang SF, 2015, SCI TRANSL MED, V7, P0, DOI 10.1126/scitranslmed.aaa5657, DOI	2015	10.51	2016	2020	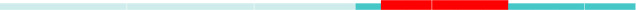
Maisel K, 2015, J CONTROL RELEASE, V197, P48, DOI 10.1016/j.jconrel.2014.10.026, DOI	2015	7.88	2016	2020	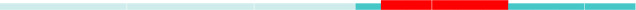
Huang Z, 2015, BIOMATERIALS, V48, P26, DOI 10.1016/j.biomaterials.2015.01.013, DOI	2015	7.35	2016	2020	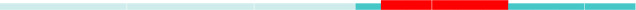
Xiao B, 2016, THERANOSTICS, V6, P2250, DOI 10.7150/thno.15710, DOI	2016	22.36	2017	2021	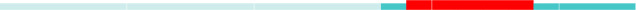
Zhang MZ, 2016, BIOMATERIALS, V101, P321, DOI 10.1016/j.biomaterials.2016.06.018, DOI	2016	12.52	2017	2021	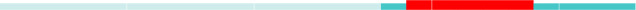
Xiao B, 2017, MOL THER, V25, P1628, DOI 10.1016/j.ymthe.2016.11.020, DOI	2017	17.91	2018	2022	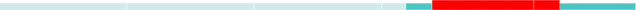
Ng SC, 2017, LANCET, V390, P2769, DOI 10.1016/S0140-6736(17)32448-0, DOI	2017	19.57	2019	2022	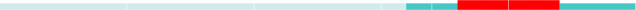
Xiao B, 2018, J CONTROL RELEASE, V287, P235, DOI 10.1016/j.jconrel.2018.08.021, DOI	2018	10.76	2019	2022	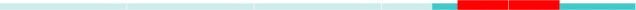
Zhang SF, 2017, NANO TODAY, V16, P82, DOI 10.1016/j.nantod.2017.08.006, DOI	2017	10.1	2019	2022	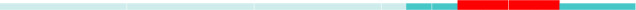
Naeem M, 2018, INT J NANOMED, V13, P1225, DOI 10.2147/IJN.S157566, DOI	2018	9.31	2019	2022	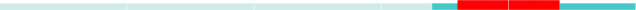
Zhang MZ, 2018, J CROHNS COLITIS, V12, P217, DOI 10.1093/ecco-jcc/jjx115, DOI	2018	11.85	2020	2022	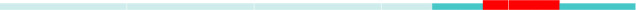
Ungaro R, 2017, LANCET, V389, P1756, DOI 10.1016/S0140-6736(16)32126-2, DOI	2017	10.68	2020	2022	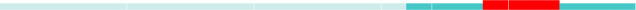
Zhang MZ, 2018, INFLAMM BOWEL DIS, V24, P1401, DOI 10.1093/ibd/izy123, DOI	2018	10.61	2020	2022	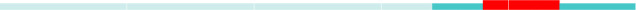
Zhang MZ, 2017, NANOMEDICINE-UK, V12, P1927, DOI 10.2217/nnm-2017-0196, DOI	2017	7.79	2020	2022	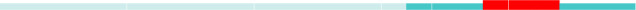
Zhang QX, 2016, BIOMATERIALS, V105, P206, DOI 10.1016/j.biomaterials.2016.08.010, DOI	2016	7.72	2020	2021	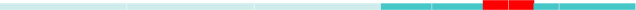
Gou SQ, 2019, BIOMATERIALS, V212, P39, DOI 10.1016/j.biomaterials.2019.05.012, DOI	2019	10.44	2021	2022	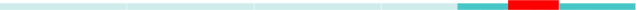
Le Berre C, 2023, LANCET, V402, P571, DOI 10.1016/S0140-6736(23)00966-2, DOI	2023	15.84	2024	2025	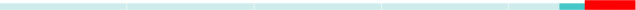
Fan X, 2023, ACS NANO, V18, P229, DOI 10.1021/acsnano.3c05732, DOI	2023	7.71	2024	2025	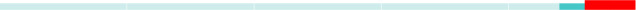

**Table 4:** The references with citation bursts from beginning to 2025.

Begin	end	Strebgth	Year	Type	Title
2024	2025	7.71	2023	Article	An Engineered Butyrate-Derived Polymer Nanoplatform as a Mucosa-Healing Enhancer Potentiates the Therapeutic Effect of Magnolol in Inflammatory Bowel Disease
2023	2025	6.26	2021	Article	Nanoparticle-assembled bioadhesive coacervate coating with prolonged gastrointestinal retention for inflammatory bowel disease therapy
2023	2025	5.65	2021	Article	Calcium pectinate and hyaluronic acid modified lactoferrin nanoparticles loaded rhein with dual-targeting for ulcerative colitis treatment
2024	2025	5.62	2023	Review	Harnessing polymer-derived drug delivery systems for combating inflammatory bowel disease
2024	2025	5.6	2023	Article	Hyaluronic acid modified oral drug delivery system with mucoadhesiveness and macrophage-targeting for colitis treatment
2023	2025	5.59	2021	Article	Colon-Targeted Adhesive Hydrogel Microsphere for Regulation of Gut Immunity and Flora
2023	2025	5.59	2022	Article	Programmable probiotics modulate inflammation and gut microbiota for inflammatory bowel disease treatment after effective oral delivery
2023	2025	5.4	2022	Article	Design of Diselenide-Bridged Hyaluronic Acid Nano-antioxidant for Efficient ROS Scavenging to Relieve Colitis
2023	2025	5.4	2022	Article	Oral administration of turmeric-derived exosome-like nanovesicles with anti-inflammatory and pro-resolving bioactions for murine colitis therapy
2023	2025	5.32	2022	Review	Nanoparticles for oral delivery: targeted therapy for inflammatory bowel disease
2024	2025	5.31	2023	Article	Orally deliverable sequence-targeted astaxanthin nanoparticles for colitis alleviation
2024	2025	5.25	2023	Article	Oral antimicrobial peptide-EGCG nanomedicines for synergistic treatment of ulcerative colitis
2024	2025	5.25	2022	Review	Novel drug delivery systems for inflammatory bowel disease
2024	2025	5.25	2022	Review	Biomaterials as therapeutic drug carriers for inflammatory bowel disease treatment
2024	2025	5.25	2022	Article	Oral nanotherapeutics based on Antheraea pernyi silk fibroin for synergistic treatment of ulcerative colitis
2024	2025	5.25	2023	Article	Oral Core-Shell Nanoparticles Embedded in Hydrogel Microspheres for the Efficient Site-Specific Delivery of Magnolol and Enhanced Antiulcerative Colitis Therapy
2023	2025	5.22	2022	Article	A nanoparticulate dual scavenger for targeted therapy of inflammatory bowel disease
2023	2025	5.06	2021	Article	Glycogen-based pH and redox sensitive nanoparticles with ginsenoside Rh2 for effective treatment of ulcerative colitis
2023	2025	5.05	2022	Article	Turmeric-derived nanovesicles as novel nanobiologics for targeted therapy of ulcerative colitis
2023	2025	5.04	2022	Article	“Dual sensitive supramolecular curcumin nanoparticles” in “advanced yeast particles” mediate macrophage reprogramming, ROS scavenging and inflammation resolution for ulcerative colitis treatment

### 3.3 Emerging trends and new developments

#### 3.3.1 Temporal variation of keyword clusters

**Figure 6:**
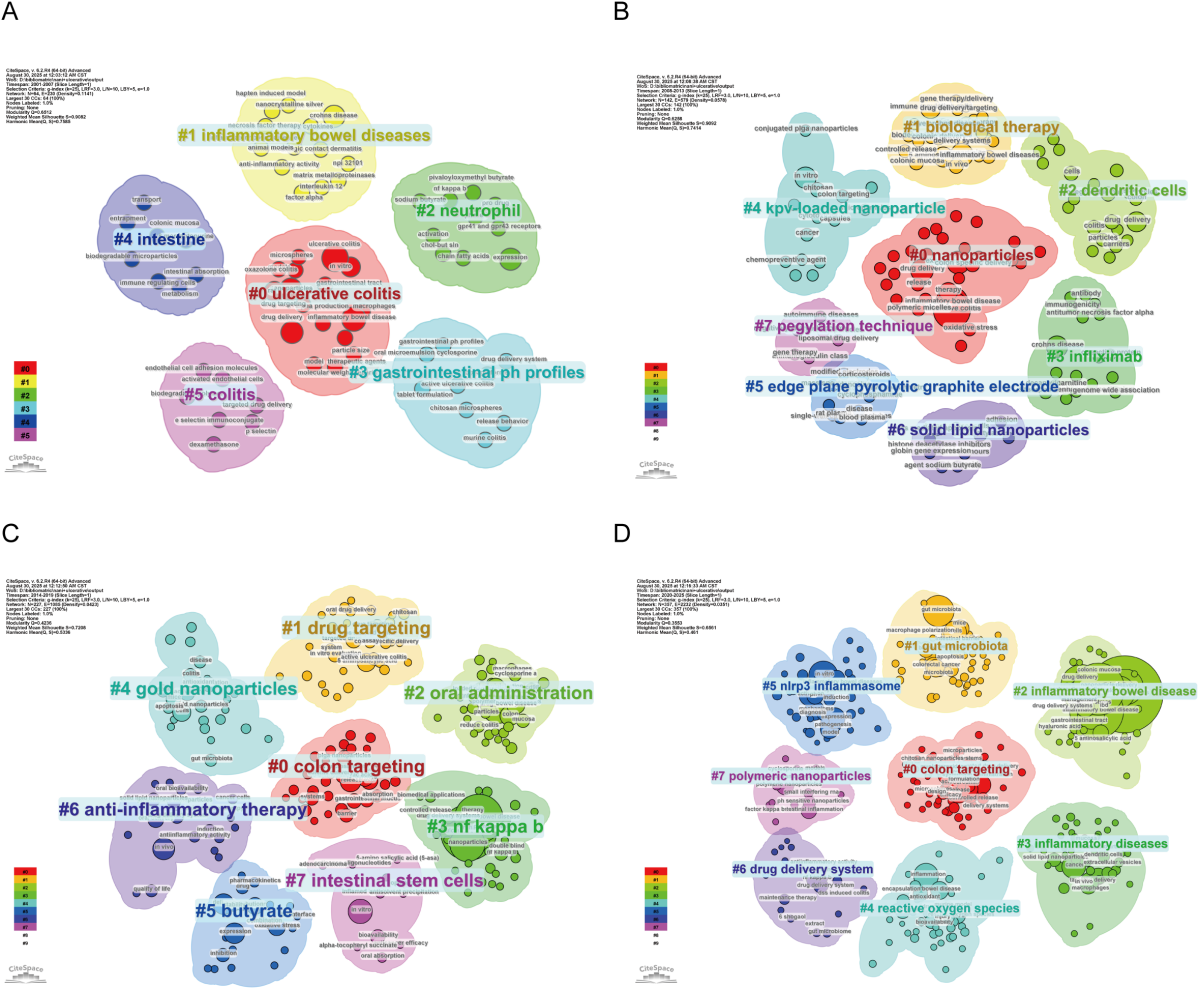
Temporal keyword co-occurrence clustering in nano-based ulcerative colitis research: **(A)** 2001–2007; **(B)** 2008–2013; **(C)** 2014–2019; **(D)** 2020–2025.

Keyword clustering was divided into four time intervals. Phase 1 (2001–2007) yielded six clusters (e.g., ulcerative colitis, neutrophils) (**Figure 6A**). Phase 2 (2008–2013) produced eight clusters (e.g., nanoparticles, dendritic cells) (**Figure 6B**). Phase 3 (2014–2019) generated eight clusters focused on colon targeting and oral administration (**Figure 6C**). Phase 4 (2020–2025) introduced new clusters including gut microbiota and reactive oxygen species (**Figure 6D**; **Supplementary Table S5**).

#### 3.3.2 Keyword alluvial flow visualization

**Figure 7:**
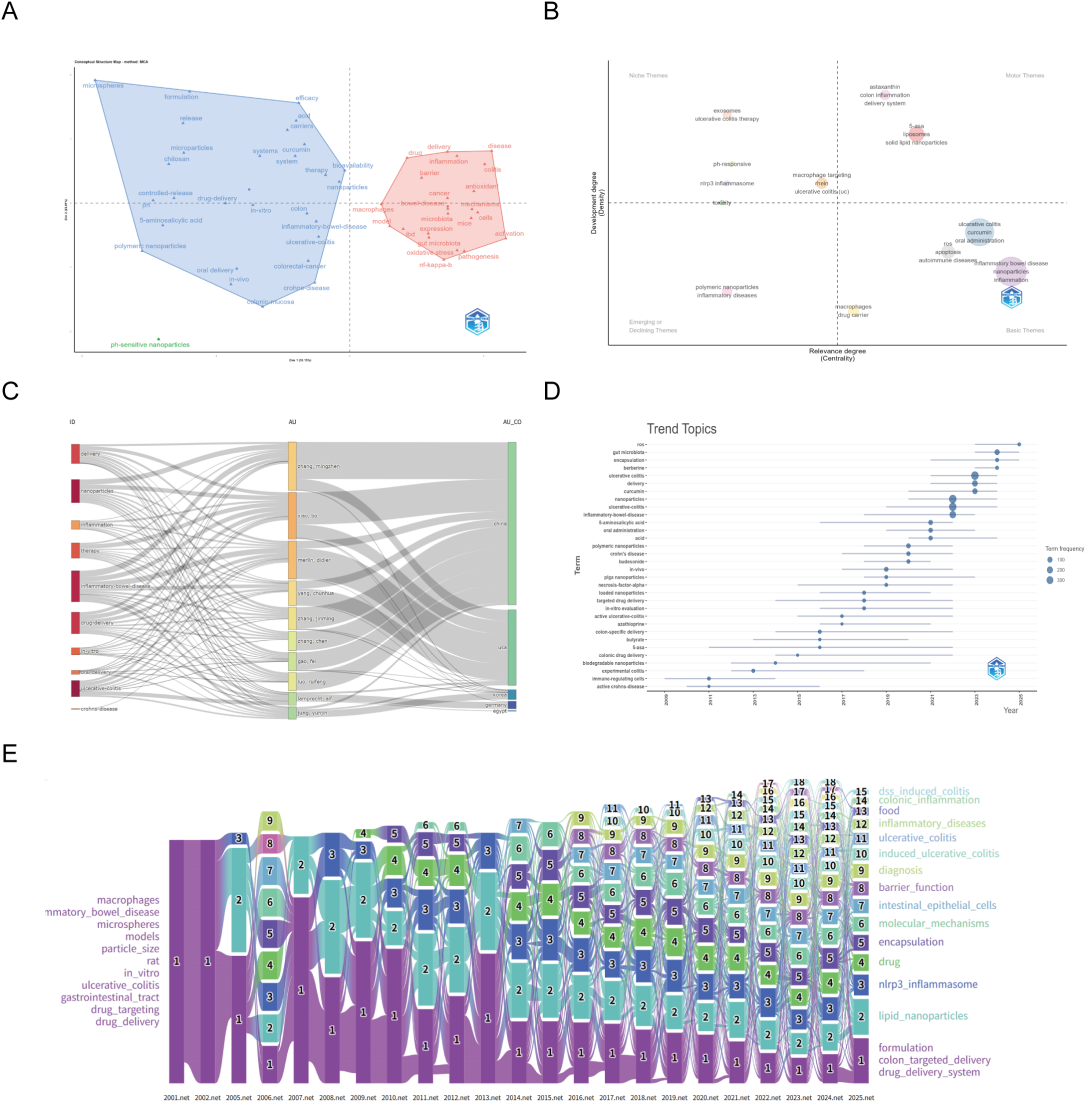
Trend analysis of theme evolution. **(A)** Multiple Correspondence Analysis, The left blue cluster represents early research topics centered on drug delivery systems, nanoparticles, and controlled release, while the right red cluster reflects later developments in biological therapies, immunomodulation, and gut microbiota. Proximity of terms indicates semantic similarity; terms far apart represent conceptually distinct research directions. **(B)** Theme Centrality Analysis, The x-axis shows reference degree (centrality), indicating how central a theme is within the citation network. The y-axis shows thematic scope, representing the breadth of related keywords. **(C)** Sankey diagram of research topic transformation, **(D)** Theme Evolution Analysis, Each dot represents a term’s frequency over time. The vertical axis lists key terms, and the horizontal axis shows years. Larger dots indicate higher occurrence. **(E)** The keyword alluvial map 2010–2022. X-axis: Time slice. Y-axis: Counting of modules.

**Figure 8:**
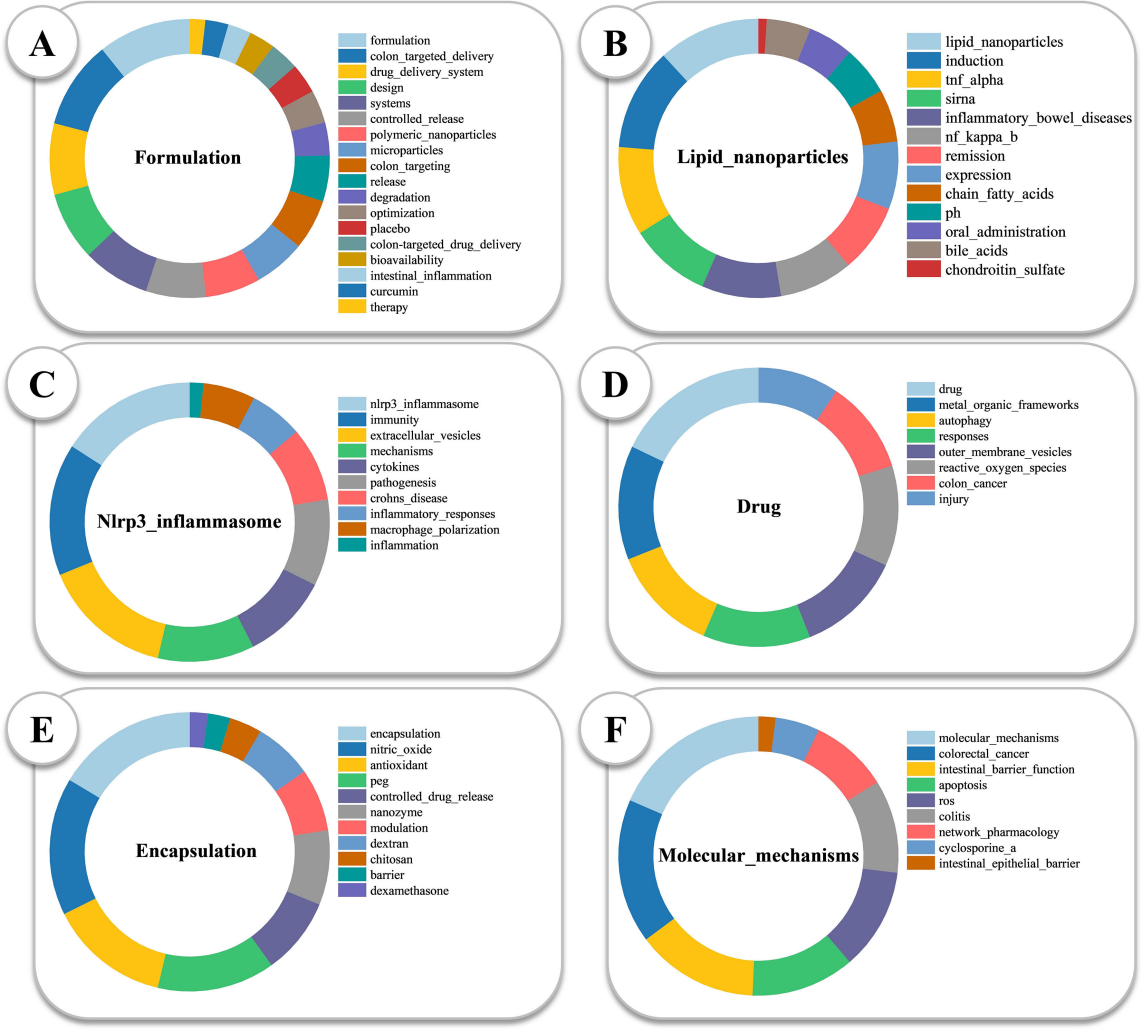
Keyword composition of the top six research modules in 2025. **(A)** Formulation:nanocarrier design and delivery strategies, including colon-targeted delivery, polymeric nanoparticles, controlled release, and microparticles. **(B)** Lipid Nanoparticles:lipid-based carriers for gene therapy and anti-inflammatory delivery, with keywords such as siRNA, chondroitin sulfate, and IBD. **(C)** NLRP3 Inflammasome:innate immune signaling, featuring cytokines, macrophage polarization, inflammation, and autophagy. **(D)** Drug:pharmacological agents and drug–mechanism interactions, including metal-organic frameworks, autophagy, and colorectal cancer. **(E)** Encapsulation:drug loading and controlled release, e.g., nitric oxide, dexamethasone, and nanosystems. **(F)** Molecular Mechanisms:cellular and molecular pathways, such as intestinal epithelial barrier, apoptosis, ROS, network pharmacology, and cyclosporine A.

Multiple Correspondence Analysis revealed thematic groupings around microparticles, drug-delivery, inflammation, and pathogenesis (**Figure 7A**). Thematic centrality mapping categorized topics into four quadrants: core themes (liposomes), specialized niches (exosomes), foundational concepts (inflammation), and emerging/declining themes (toxicity) (**Figure 7B**). The Sankey diagram showed strong China–U.S. collaboration in oral targeted therapy (**Figure 7C**). Thematic evolution shifted from colon-specific delivery to biodegradable nanocarriers and microbiota modulation (**Figure 7D**). The keyword alluvial map 2010–2022 is shown in **Figure 7E**. Six keyword modules with highest annual traffic are shown in **Figure 8**; top five are listed in **Supplementary Table S6**.

#### 3.3.3 Timeline visualization of references

**Figure 9:**
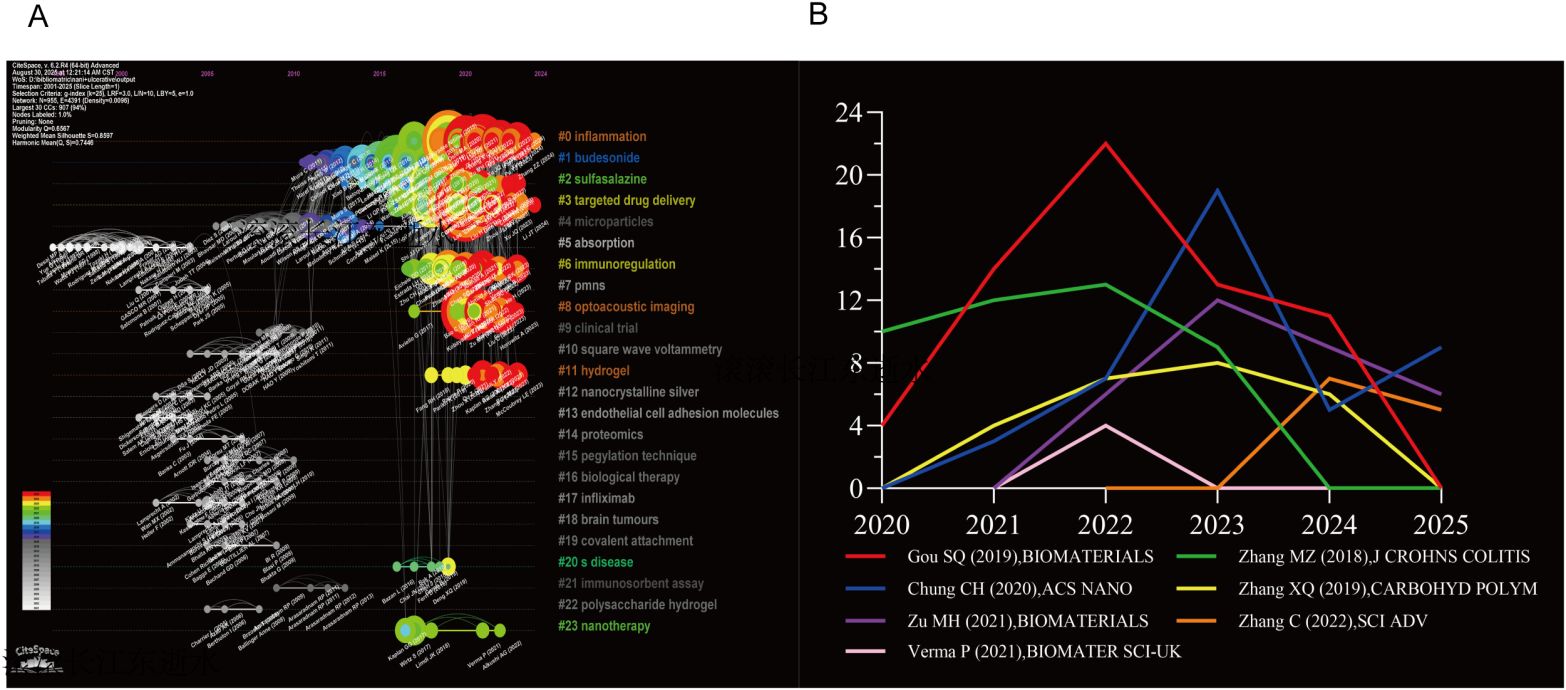
The reference cluster map. **(A)** Timeline of cited literature. **(B)** Citation frequency distribution of the burst citations, X-axis:year, Y-axis:citation frequency.

The citation timeline comprised 14 clusters. Persistent topics included Budesonide, Microparticles, and disease-related terms (#1, #4, #20) (**Figure 9A**). Emerging topics included inflammation, targeted drug delivery, and nanotherapy. Classic papers continued to be cited (**Figure 9B**); detailed cluster data are in **Supplementary Table S7**.

### 3.4 Analysis of publication trends and geographical distribution based on combined data from two databases

**Figure 10:**
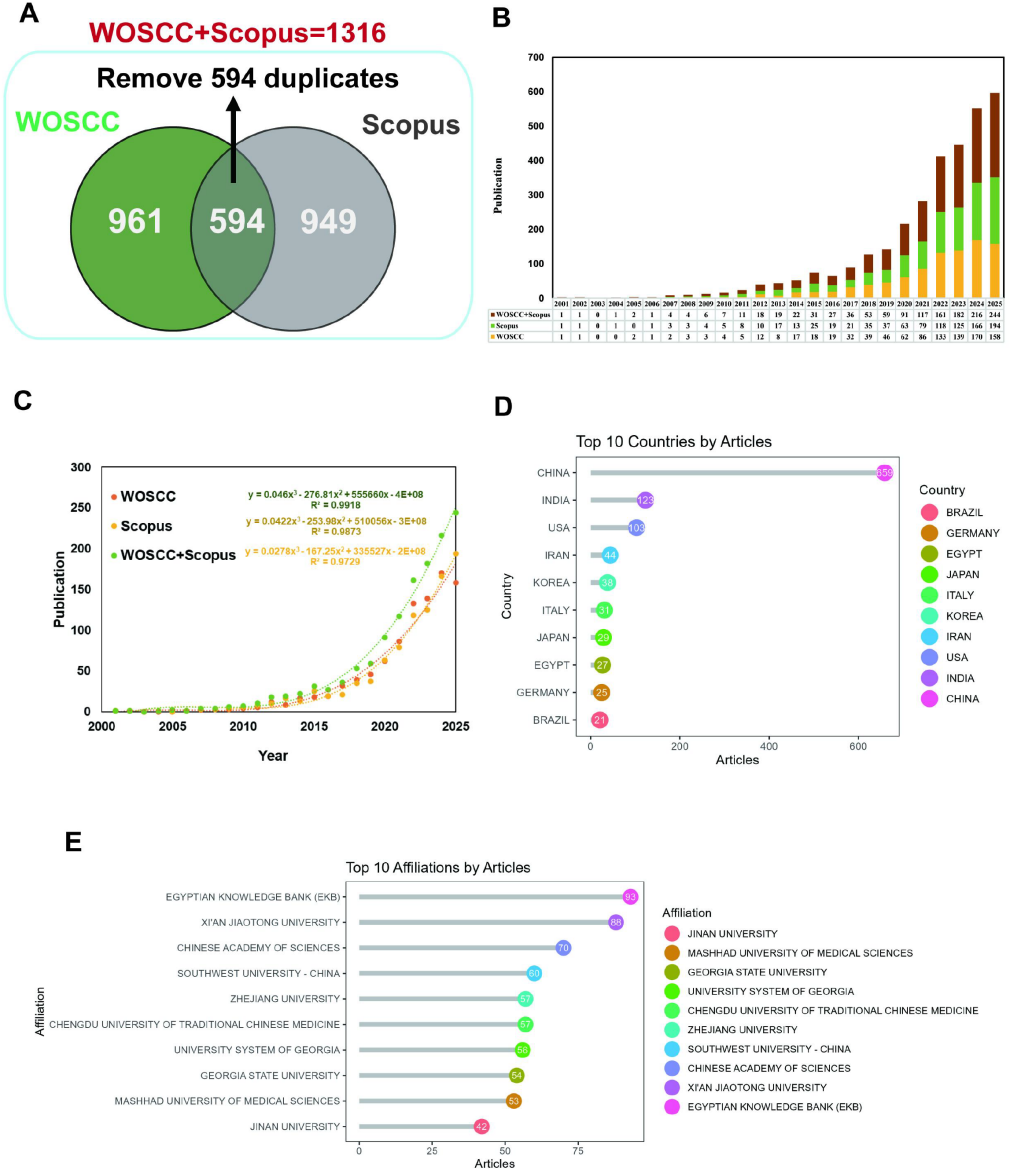
Bibliometric analysis based on integrated data from Web of Science Core Collection (WOSCC) and Scopus. **(A)** Dataset integration: After deduplication, 1,316 unique publications were identified from the combined WOSCC (961 records) and Scopus (949 records), with 594 overlapping entries removed. **(B)** Distribution of literature sources: Publications were primarily sourced from journals in nanomedicine, pharmacology, and gastroenterology, reflecting the field’s interdisciplinary scope. **(C)** Annual publication trend: Annual output remained below 10 before 2005, increased steadily after 2010, and exceeded 100 in 2024; nonlinear regression confirmed exponential growth (combined R² = 0.9918). **(D)** Top 10 productive countries: China led with 659 publications, followed by India (123), the United States (103), Iran (44), South Korea (38), Italy (31), and Egypt (27). **(E)** Top 10 productive affiliations: Egyptian Knowledge Bank ranked first (93 publications), followed by Xi’an Jiaotong University (88); the majority of top institutions were from China.

After deduplication, 1,316 publications were identified from WOSCC and Scopus combined (961 from WOSCC, 949 from Scopus, 594 overlapping) (**Figure 10A**; **Supplementary Table S2**). Annual output was <10 before 2005, rose steadily after 2010, and exceeded 100 in 2024 (**Figure 10B**). Nonlinear regression showed exponential growth (combined R² = 0.9729) (**Figure 10C**). China led in output (659), followed by India (123) and the U.S. (103); Iran (44), South Korea (38), Italy (31), and Egypt (27) also ranked highly (**Figure 10D**). Institutionally, Egyptian Knowledge Bank (93), Xi’an Jiaotong University (88), and other Chinese institutions dominated the top ten (**Figure 10E**).

### 3.5 Parallel validation of collaborative networks based on two databases

To assess the reliability and consistency of bibliographic databases in mapping academic collaboration networks, collaboration networks at the author, country, and institution levels were constructed using WOSCC and Scopus. Co-occurrence relationships of high-output authors, countries, and institutions were visualized using VOSviewer, validating the stability and complementarity of the two databases in revealing collaborative network structures.

**Figure 11:**
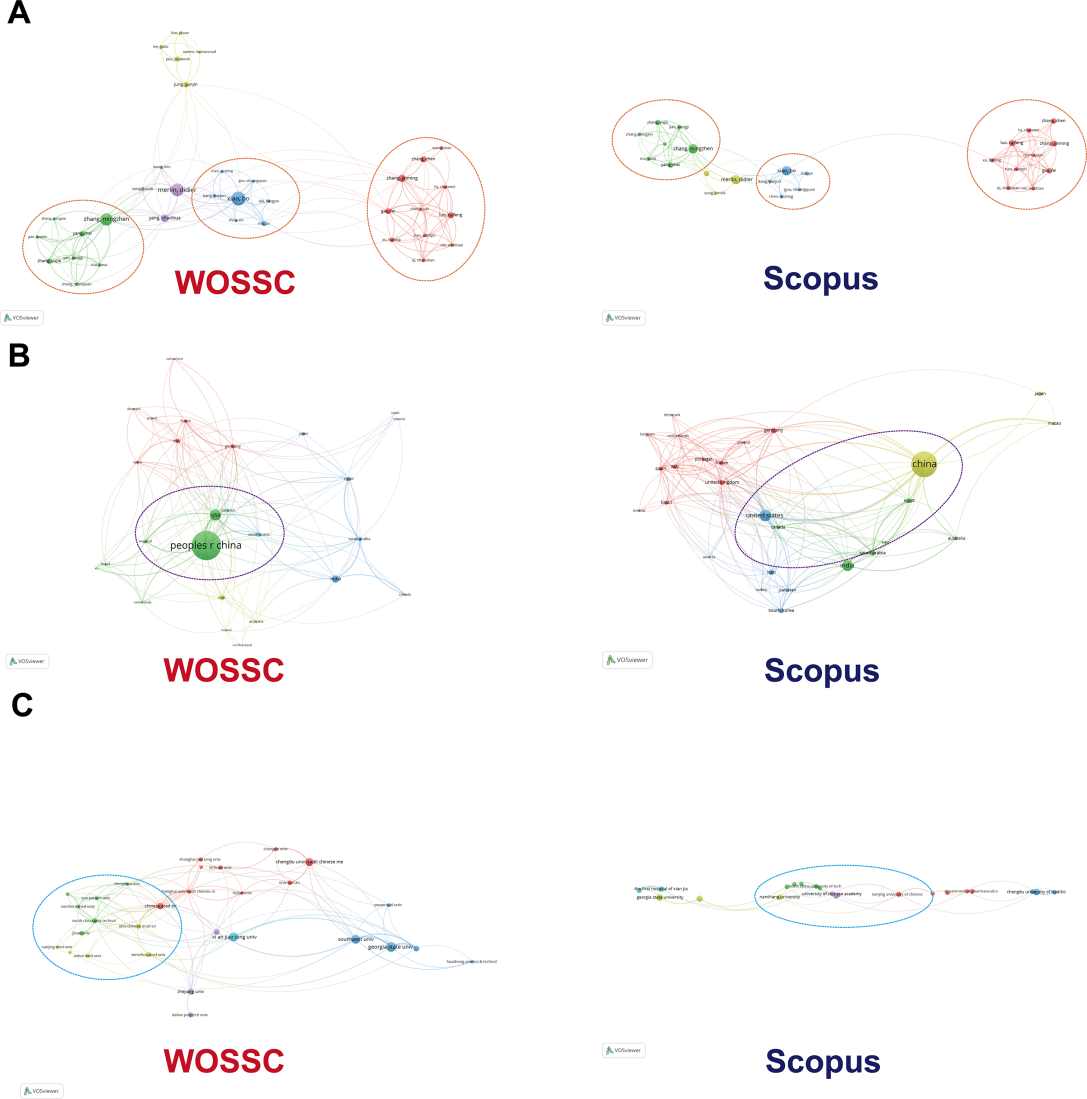
Parallel bibliometric comparison of Web of Science Core Collection (WOSCC) and Scopus datasets across multiple analytical dimensions. **(A)** Author level; **(B)** Country level; **(C)** Institutional level.

In the author collaboration network, both databases identify several tightly connected sub-communities, indicating core research teams in the field. Chinese author groups (e.g., “Zhang,” “Chen,” “Li”) appear in both networks, reflecting a strong domestic collaboration network. However, international scholars such as “Smith” and “Johnson” exhibit different connection strengths and placements across the two databases. Scopus displays a more dispersed international network, while WOSCC emphasizes domestic cooperation. This disparity may stem from differences in journal coverage, language preferences, and citation standards (**Figure 11A**). While both databases align in identifying major collaboration groups, slight discrepancies in localized collaborations suggest the value of combining data from multiple sources.

At the country level (**Figure 11B**), both WOSCC and Scopus place China at the center, reflecting its dominant role in global collaborations. WOSCC shows a “central-radiation” structure, with strong ties to the U.S., India, and Iran. Scopus, however, features a more complex network, emphasizing connections not only with Western countries but also with emerging nations in the Middle East and Africa, particularly Egypt and Saudi Arabia. The United States appears more centrally in Scopus, while its connections in WOSCC are less pronounced, likely due to Scopus’s broader inclusivity of non-English journals and regional publications.

At the institutional level (**Figure 11C**), WOSCC highlights a tightly interconnected network of Chinese institutions, with Xi’an Jiaotong University at the center. In contrast, Scopus presents a more flattened network, focusing on multinational collaborations, such as those involving the Chinese Academy of Sciences and the University of Georgia. Scopus also highlights the Egyptian Knowledge Bank, a connection not as strongly represented in WOSCC, suggesting that Scopus is better at capturing the collaborative involvement of regional knowledge platforms.

While both databases align in identifying key research hubs (e.g., China, Xi’an Jiaotong University, Zhang), they differ in their emphasis on international collaboration and the diversity of collaborative patterns. Scopus captures a broader international scope, especially among developing countries, while WOSCC focuses on stable collaborations among traditional research powerhouses. Despite these differences, the core collaboration patterns remain consistent, and the parallel analysis of both databases enhances the reliability and comprehensiveness of the results, offering a more complete understanding of global research collaboration dynamics.

## 4 Discussion

### 4.1 Current status of nanotherapy in UC based on bibliometric analysis

Our bibliometric analysis reveals a notable advancement in research on nanotherapy for UC, particularly between 2001 and 2025, with a significant increase in the number of publications. From 2001 to 2013, the volume of literature remained relatively modest. However, starting in 2014, the number of studies increased sharply, particularly in recent years, marking a phase of rapid development in this field. This research demonstrates the highly collaborative nature of the discipline, with strong networks of authors, institutions, countries, and research teams providing substantial support for its further advancement. Notably, countries such as China, the United States, India, and South Korea, along with prominent research institutions such as the Chinese Academy of Sciences and the University System of Georgia, have played a pivotal role in fostering global cooperation within this area.

In terms of research hotspots, changes in keywords over the past 16 years reflect the interdisciplinary nature of nanotherapy in UC treatment. For instance, “gut microbiota” has shown a significant surge between 2023 and 2025, underscoring the growing importance of the gut microbiome in UC therapy. Additionally, the emergence of terms such as “immune modulation” and “polymeric nanoparticles” highlights the integration of immune system regulation and drug delivery technologies in the development of novel nanomaterials. Furthermore, strategies for targeted delivery in UC, such as colon-specific drug delivery and the restoration of intestinal barrier function, have become focal points of research, illustrating the potential of nanotechnology in precise disease treatment.

By analyzing keyword flow diagrams and citation networks, we observe a shift in the application of nanoparticles in UC treatment from basic research to clinical translation. Throughout this process, drug delivery systems, intestinal targeting therapies, and the precise modulation of the inflammatory microenvironment have emerged as key topics.

### 4.2 Mechanisms of nanotherapy in UC

UC is a prototypical chronic inflammatory bowel disease, characterized by prolonged inflammation that not only damages intestinal tissues but also compromises intestinal barrier function and induces immune system abnormalities (13). Traditional therapies often face limitations in efficacy and significant side effects. However, nanotherapy, with its excellent targeting capabilities, controlled release mechanisms, and multifaceted modes of action, presents promising therapeutic potential (14). As nanotechnology advances, nanoparticles, as drug carriers, not only enhance drug bioavailability and release profiles but also modulate inflammation, repair the intestinal barrier, and regulate the immune microenvironment, offering innovative solutions for UC treatment. This section summarizes key mechanisms of nanotherapy in UC treatment, detailing how they interact with the pathophysiological features of UC to provide more efficient and precise therapeutic strategies.

#### 4.2.1 Targeted delivery

The targeted delivery capacity of nanoparticles is one of the core advantages of nanotherapy in treating UC (15). UC’s pathological features are often associated with localized intestinal inflammation and increased vascular permeability, allowing drugs to penetrate more easily into inflamed regions. Exploiting this physiological characteristic, nanoparticles, through enhanced permeability and retention (EPR) effects, can selectively accumulate in diseased areas, thereby significantly increasing local drug concentrations (16). Furthermore, factors such as nanoparticle size, surface charge, and surface modifications notably influence their distribution and accumulation within the colon. By precisely designing these properties, efficient drug delivery to inflammatory sites can be achieved. For example, nanoparticles modified with the natural polysaccharide euphorbia polyphenol (EUP) have shown to improve colon tissue, intestinal barrier function, inflammatory factors, and gut microbiota composition (17). Additionally, lactoferrin (LF) nanoparticles modified with calcium pectate (CP) and hyaluronic acid (HA) effectively deliver rhizoma radix (RH) while mitigating inflammation via inhibition of the TLR4/MyD88/NF-κB signaling pathway, promoting colonic repair (18). Similarly, curcumin and Prussian blue-modified CCM-CoFe PBA nanoparticles have effectively alleviated DSS-induced UC symptoms in mice (19).

#### 4.2.2 Immune modulation

The onset and progression of UC are closely associated with the aberrant activation of the immune system (20). Chronic inflammation can lead to excessive immune cell activation and abnormal cytokine secretion, further exacerbating colonic damage (21). For example, hyaluronic acid-modified poly(lactic-co-glycolic acid) (HA-PLGA) nanoparticles have demonstrated effective targeting and delivery of bilirubin for UC treatment, improving intestinal morphology, barrier function, and immune response regulation (22). Moreover, flavonoid compounds such as quercetin restore NCR-ILC3/NCR+ILC3 balance, promote IL-22 secretion, and repair intestinal barriers, significantly alleviating UC symptoms in mice (23). These findings suggest that nanoparticles, through their interaction with the immune system, can modulate immune responses and reduce persistent inflammation. Various types of nanoparticles, such as polymeric and lipid nanoparticles, can selectively modulate immune response intensity through interactions with surface receptors on immune cells like macrophages and dendritic cells. For example, polymeric nanoparticles can promote immune tolerance and reduce the release of pro-inflammatory cytokines, thus alleviating chronic UC inflammation (24–26). Lipid nanoparticles, on the other hand, modulate T-cell function, induce anti-inflammatory responses, and further mitigate intestinal inflammation (27, 28). Additionally, nanoparticles can carry immune-modulating molecules, such as anti-inflammatory cytokines and antioxidants, for targeted delivery, effectively inhibiting unnecessary immune reactions and enhancing therapeutic efficacy (29).

#### 4.2.3 Antioxidant effects

Oxidative stress is a key pathogenic factor in the progression of UC (30). The excessive production of reactive oxygen species (ROS) not only directly damages colonic cells but also activates multiple inflammatory signaling pathways, further exacerbating intestinal inflammation (31). Nanocarriers loaded with antioxidants can effectively scavenge excess ROS at inflammatory sites, reducing oxidative stress (32). For example, nanoparticles loaded with antioxidants such as vitamin C and curcumin can directly combat oxidative stress at inflammation sites through targeted delivery, alleviating colonic cell damage (33). The use of antioxidant nanoparticles not only improves the pathological state of UC but also promotes the repair of colonic epithelial cells, aiding in the restoration of intestinal function (34).

#### 4.2.4 Intestinal barrier repair

The disruption of the intestinal barrier is a hallmark pathological feature of UC (35). Damage to the intestinal barrier not only increases gut permeability but may also trigger microbial imbalance and immune system dysregulation, further exacerbating inflammation (36). Nanoparticles, by delivering repair agents to targeted sites, can aid in the restoration of the intestinal barrier and improve colon function. Natural compounds such as hyaluronic acid and curcumin have been incorporated into nanoparticle carriers for targeted delivery to inflammation sites, repairing damaged intestinal epithelial cells and restoring barrier integrity (37). Furthermore, nanoparticles can modulate the gut microbiome’s composition, restoring microbial balance and promoting the repair of the intestinal immune barrier (38). Through this dual mechanism, nanoparticles not only enhance drug efficacy but also improve the intestinal barrier’s self-repair capacity.

### 4.3 Clinical applications of nanotherapy in UC

Nanotherapy is emerging as a promising approach for ulcerative colitis (UC), offering targeted drug delivery, biocompatibility, and tunable design—advantages over traditional therapies hampered by limited efficacy and side effects. With growing translation from preclinical studies to clinical trials, its clinical potential is increasingly evident.

#### 4.3.1 Targeted drug delivery systems

Targeted drug delivery is a key nanotherapeutic strategy for UC, in which engineered nanoparticles enable site-specific drug release, thereby enhancing efficacy and reducing systemic toxicity. Hyaluronic acid–modified polymeric nanoparticles have been used to co-deliver curcumin and siCD98 to inflamed colonic tissue, resulting in increased local drug accumulation and enhanced anti-inflammatory effects (39, 40). These systems enable localized drug release at inflamed sites, reducing systemic exposure and toxicity. Ligand-functionalized nanoparticles are now advancing into clinical trials, offering more precise targeting, increased drug accumulation at disease sites, and improved therapeutic efficacy (18).

#### 4.3.2 Personalized and multifunctional nanotherapeutic strategies

Personalized and multifunctional nanotherapy is an emerging strategy for UC treatment, aiming to address interindividual heterogeneity in disease pathology and immune status. By integrating patient-specific factors—such as gut microbiota composition, immune responses, and inflammation severity—nanoparticles can be rationally designed for precision drug delivery and immune regulation. Functionally engineered and surface-modified nanoparticles enable adaptive therapeutic responses, alleviating inflammation while limiting immune overactivation.

Meanwhile, multifunctional nanoplatforms that combine targeted drug delivery with antioxidant, immunomodulatory, and intestinal barrier–repair functions offer a more comprehensive intervention across UC pathogenic pathways. Co-delivery of anti-inflammatory agents (e.g., curcumin) and antioxidants (e.g., superoxide dismutase) suppresses inflammation, scavenges excess ROS, and promotes mucosal healing, thereby reducing drug burden and improving therapeutic efficacy (41, 42).

#### 4.3.3 Clinical trials and practical applications

Currently, clinical trials based on nanomaterials are gradually underway, particularly in oral nanodrug formulations. Researchers aim to enhance drug bioavailability at inflammation sites through nanoparticles, thereby improving therapeutic efficacy. Novel formulations such as hyaluronic acid-modified nanoparticles and targeted nanoparticles have yielded preliminary results in clinical research for UC, especially in oral administration, offering patients more convenient treatment options (43). Clinical studies have shown that oral nanovitamin D supplementation can significantly reduce disease activity and severity in patients with active UC (44). As clinical trials progress, nanotherapy is poised to become a new, alternative treatment option, particularly for chronic inflammatory diseases such as UC. In the future, nanodrug delivery systems will be integrated with personalized treatment strategies, providing patients with safer and more effective therapeutic options.

### 4.4 Comparison with conventional and emerging therapies

Although aminosalicylates, corticosteroids, immunosuppressants, and biologics have long been the cornerstone therapies for ulcerative colitis (UC), these conventional treatments exhibit certain limitations regarding efficacy, systemic side effects, and patient adherence (45). For instance, corticosteroids, while effective in suppressing inflammation, may lead to metabolic disturbances and an increased risk of infections with prolonged use. Immunosuppressants and biologics, though highly efficacious, demonstrate variable responses due to patient disease phenotype heterogeneity and may induce systemic immune suppression (46).

In contrast, nanotherapy has shown considerable advantages in the treatment of UC. By enabling targeted delivery to inflamed colonic regions, nanoparticles can enhance the local drug concentration while minimizing systemic exposure, thereby reducing the risk of toxicity (47). Moreover, multifunctional nanoplatforms can simultaneously regulate multiple pathogenic pathways, including immune dysregulation, oxidative stress, and intestinal barrier damage, which are often beyond the scope of single conventional drugs (48).

Compared to emerging therapies such as gut microbiota transplantation or small-molecule inhibitors, nanotherapy offers greater flexibility in drug design, controlled release, and multifunctional integration. Therefore, nanotherapy holds promise as a powerful complement or alternative to traditional drugs and emerging therapies, addressing the multifaceted limitations of current treatments.

### 4.5 Prospects for clinical translation

Nanotherapeutic approaches have demonstrated early momentum toward clinical translation in ulcerative colitis (UC). Available evidence, primarily derived from early-phase and exploratory studies, indicates that orally administered nanoformulations may enhance local bioavailability, improve colon-specific drug delivery, and maintain acceptable safety profiles. Nonetheless, current support for clinical application remains limited, as most findings originate from preliminary investigations rather than large-scale, confirmatory clinical trials.

Several barriers continue to impede further translation. These include difficulties in achieving controllable and reproducible large-scale manufacturing, insufficient long-term assessment of biocompatibility and safety, incomplete clarification of regulatory approval pathways, and challenges in aligning nanotherapeutic strategies with existing treatment algorithms and precision medicine frameworks.

Progress in this field will therefore require coordinated multidisciplinary efforts, supported by rigorous preclinical validation and the establishment of standardized, reproducible evaluation criteria and translational frameworks. Future studies, conducted under stringent safety and efficacy assessment, may investigate the integration of nanodelivery systems with patient-specific disease characteristics, including gut microbiota composition, inflammatory biomarkers, and immune phenotypes, to improve therapeutic precision and controllability. In parallel, multifunctional nanoplatforms that integrate drug delivery with modulation of inflammatory or immune pathways may provide complementary therapeutic options for UC; however, their clinical relevance must be established through systematic, well-designed clinical investigations.

### 4.6 Limitations and implications

This study is subject to methodological limitations inherent to bibliometric analyses. First, data were drawn exclusively from the Web of Science Core Collection and Scopus and truncated on July 1, 2025, excluding publications from July to December 2025, which may underrepresent the most recent advances in rapidly evolving areas such as gut microbiota modulation and exosome-based nanotherapies. Moreover, the reliance on only these two databases may omit relevant studies indexed in other major sources such as PubMed or Embase, potentially narrowing the scope of coverage. Second, both databases predominantly index English-language, peer-reviewed journal articles, introducing potential language and source biases. This limitation may exclude valuable contributions published in non-English journals, conference proceedings, preprints, grey literature, or regionally focused research that does not conform to mainstream publication channels. Third, all thematic and structural insights were derived from algorithmic metrics, including co-citation, keyword co-occurrence, and burst detection, which reflect quantitative visibility rather than scientific quality, clinical relevance, or methodological rigor. Bibliometric approaches, by design, cannot assess the validity, reproducibility, or translational potential of individual studies. Fourth, keyword standardization and indexing rely heavily on automated database tagging, which may introduce semantic inconsistencies or fail to capture nuanced conceptual variations across disciplines. Additionally, author and institutional disambiguation was performed automatically, which may conflate distinct researchers or misattribute affiliations, particularly within large collaborative networks.

Despite these constraints, the dual-database approach enhances coverage and cross-validation, and the convergence of findings across multiple analytical dimensions strengthens the robustness of the results. To address these limitations in future work, we recommend complementing bibliometric mapping with qualitative systematic reviews or mixed-methods approaches that integrate expert appraisal of methodological quality and contextual interpretation of emerging trends. Overall, the resulting map of global research activity in nano-based UC therapies provides a transparent, data-driven foundation to inform future strategic planning and foster interdisciplinary collaboration.

## 5 Conclusion

Our study unveils the rapid progression of research into nano-based therapies for UC, emphasizing the interdisciplinary nature and burgeoning potential of this field. Since the early 2000s, the volume of related literature has seen a marked increase, particularly from 2014 onwards, reflecting the growing interest and advancing application of nanotechnology in UC treatment. Research hotspots such as gut microbiota, immune modulation, and targeted drug delivery have emerged as central themes, with contemporary studies increasingly focusing on innovative strategies such as multifunctional nanoplatforms and personalized therapeutic approaches. The mechanisms of nanotherapy, encompassing targeted delivery, immune modulation, antioxidant effects, and intestinal barrier repair, are progressing rapidly, offering new avenues for more effective and precise treatment of UC. Moreover, the shift from foundational research to clinical application is becoming more evident, with nanomaterials providing promising solutions to overcome the limitations of conventional therapies. The ongoing advancement of clinical trials and the integration of personalized nanotherapy strategies signal a transformative shift in the treatment paradigm of UC. As research continues to evolve, nanotherapy holds the potential to become a cornerstone in UC management, paving the way for more effective, individualized, and minimally invasive treatment options.

## Data Availability

The original contributions presented in the study are included in the article/**Supplementary Material**. Further inquiries can be directed to the corresponding author.
