# Supplementary material for: Structural and temporal dynamics of nano-based therapies in ulcerative colitis: history, hotspots, and emerging trends
Source: Front Immunol. 2026 Feb 18;17:1739037. doi: 10.3389/fimmu.2026.1739037 (PMC12957217; doi:10.3389/fimmu.2026.1739037)
Supplement: Supplementary file 1 [file DataSheet1.pdf]

Supplementary materials

Table S1 The specific search strategies, search dates, and inclusion criteria used for the Web of Science Core Collection (WoSCC) and Scopus databases.

Database	Search Strategy	Search Date	Filtering Conditions
WOSCC	TS = (nanodot OR nanoparticle OR nanomaterial OR nanotube OR nanosheet* OR "quantum dot*" OR nanofiber* OR nanosphere* OR nanorod* OR nanowire* OR nanocrystal* OR nanocomposite* OR nanodevice* OR nanocluster* OR nanotechn* OR nanocarrier* OR nanoliposome* OR nanoemulsion* OR nanoconjugate* OR nanogels* OR nanodiamond* OR nanosilver* OR nanopore* OR nanomicell* OR nano size* OR nanomedicine*) AND ((TS=("ulcerative colitis*") OR TS=("Colitis, Ulcerative*") OR TS=("Colitis Gravis*") OR TS=("Idiopathic Proctocolitis*") OR TS=("Inflammatory Bowel Disease, Ulcerative Colitis Type*")))	The literature search was conducted on July 1, 2025.	English-language publications from January 1, 2001, to July 1, 2025, including Articles and Review Articles.
Scopus	(TITLE-ABS-KEY(("nanodot*" OR "nanoparticle*" OR "nanomaterial*" OR "nanotube*" OR "nanosheet*" OR "quantum dot*" OR "nanofiber*" OR "nanosphere*" OR "nanorod*" OR "nanowire*" OR "nanocrystal*" OR "nanocomposite*" OR "nanodevice*" OR "nanocluster*" OR "nanotechn*" OR "nanocarrier*" OR "nanoliposome*" OR "nanoemulsion*" OR "nanoconjugate*" OR "nanogels*" OR "nanodiamond*" OR "nanosilver*" OR "nanopore*" OR "nanomicell*" OR "nano size*" OR "nanomedicine*")) AND TITLE-ABS-KEY(("ulcerative colitis*" OR "Colitis, Ulcerative*" OR "Colitis Gravis*" OR "Idiopathic Proctocolitis*" OR "Inflammatory Bowel Disease,	The literature search was conducted on July 1, 2025.	English-language publications from January 1,2001, to July 1, 2025, including Articles and Review Articles.

Ulcerative Colitis Type*"))) AND (LIMIT-TO (DOCTYPE,"ar") OR LIMIT-TO (DOCTYPE,"re")) AND (LIMIT-TO (LANGUAGE,"English")) AND PUBYEAR > 2001

Table S2 Comparative Analysis of Bibliometric Indicators Based on WOSCC and Scopus Databases (2001-2025)

**Description**	**WOSCC**	**Scopus**	**WOSCC+Scopus**
Timespan	2001:2025	2001:2025	2001:2025
Sources (Journals, Books, etc)	315	350	437
Documents	961	949	1315
Annual Growth Rate %	23.48	5.7	5.7
Document Average Age	3.61	3.56	3.71
Average citations per doc	34.71	44.03	38.26
Keywords Plus (ID)	1949	10600	9016
Author's Keywords (DE)	2200	2291	3005
Authors	5599	5361	4539
Authors of single-authored docs	5	12	15
Single-authored docs	5	16	19
Co-Authors per Doc	7.71	7.2	7.3
International co-authorships %	25.18	25.32	18.4
article	751	663	918
review	210	286	398

Table S3 The top 10 countries, institutions and authors for frequency of co-occurrence

CoF	Year	Country	CoF	Year	Institution	CoF	Citation	Author
556	2012	CHINA	46	2017	Chinese Academy of Sciences	30	2419	xiao, bo
144	2005	USA	42	2010	University System of Georgia	28	3363	merlin, didier
78	2009	INDIA	41	2012	Georgia State University	27	2211	zhang, mingzhen
40	2012	IRAN	35	2016	Southwest University - China	17	596	yang, chunhua
39	2014	SOUTH KOREA	34	2020	Xi'an Jiaotong University	16	806	zhang, jinming
37	2001	GERMANY	33	2016	Egyptian Knowledge Bank (EKB)	14	572	luo, ruifeng
35	2006	ITALY	33	2019	Chengdu University of Traditional Chinese Medicine	14	657	zhang, chen
34	2018	SAUDI ARABIA	28	2010	Veterans Health Administration (VHA)	13	618	gao, fei
33	2016	EGYPT	28	2010	US Department of Veterans Affairs	13	457	lamprecht, alf
28	2002	JAPAN	22	2015	Atlanta VA Health Care System	12	713	jung, yunjin

CoF: Co-occurrence frequency. Year:Year of first collaboration appearance

Table S4 The top 20 subject categories and keywords burst with a burst period from beginning to 2025

**Subject category bursts**					**Keywords bursts**				
Begin	End	Strength	Year	Entity	Begin	End	Strength	Year	Entity
2024	2025	2.28	2010	PHYSICS, CONDENSED MATTER	2023	2025	8.17	2019	gut microbiota
2024	2025	1.73	2016	FOOD SCIENCE & TECHNOLOGY	2023	2025	2.83	2023	macrophage polarization
2024	2025	1.39	2011	CHEMISTRY, APPLIED	2023	2025	2.77	2018	mechanisms
2024	2025	0.91	2022	AGRICULTURE, MULTIDISCIPLINARY	2024	2025	2.77	2024	exosm
2024	2025	0.63	2018	INSTRUMENTS & INSTRUMENTATION	2023	2025	2.74	2023	hyaluronic acid
2024	2025	0.51	2010	PHYSICS, APPLIED	2023	2025	2.44	2019	maintenance therapy
2024	2025	0.5	2024	MATERIALS SCIENCE, COATINGS & FILMS	2024	2025	2.38	2024	sirna
2024	2025	0.5	2024	NEUROSCIENCES	2024	2025	2.18	2019	stability
2024	2025	0.5	2024	CHEMISTRY, INORGANIC & NUCLEAR	2024	2025	2.16	2022	targeted therapy
2024	2025	0.41	2017	ONCOLOGY	2024	2025	2.12	2024	mesalazine
-	-	-	-	-	2024	2025	2.12	2024	immune responses
-	-	-	-	-	2023	2025	2.04	2009	microparticles
-	-	-	-	-	2023	2025	1.99	2023	food
-	-	-	-	-	2024	2025	1.98	2024	epithelial barrier
-	-	-	-	-	2024	2025	1.98	2024	intestinal flora
-	-	-	-	-	2023	2025	1.91	2023	mucus
-	-	-	-	-	2023	2025	1.91	2023	metabolites
-	-	-	-	-	2024	2025	1.88	2013	barrier function
-	-	-	-	-	2022	2025	1.88	2022	reactive oxygen species

-	-	-	-	-	2023	2025	1.84	2022	health

Bigin: the burst’ beginning year, End: the burst’ ending year, Strength: the burst’ strength index, Year: the first appearance time, Entity: the term.

Table S5 Summary of keyword clusters for the most recent stage(2020-2025)

ClusterID	Size	Silhouette	Average Year	Label (LLR)	Representative keywords
0	71	0.659	2021	colon targeting	ulcerative colitis; yeast cell wall microparticles; mucosal repair; expression; vivo evaluation | inflammatory bowel disease; colon delivery; dependent systems; particle size; ph-responsive polymers
1	58	0.573	2022	gut microbiota	ulcerative colitis; drug delivery; mucus; therapy; cells | inflammatory bowel disease; precancerous lesions; colitis-associated colon cancer; dietary supplement; microbiotametabolite regulation
2	53	0.658	2021	inflammatory bowel disease	ulcerative colitis; alginate; mucosal delivery; chitosan; precise therapy | inflammatory bowel disease; targeted drug delivery; pathological features; small interfering rna delivery; cationic polymers
3	49	0.601	2022	inflammatory diseases	inflammatory bowel disease; extracellular vesicles; exosome-like nanoparticles; cell membranes; intestinal epithelial cells | ulcerative colitis; colorectal cancer; nano-drug delivery; cell membranes; intestinal epithelial cells
4	45	0.697	2022	reactive oxygen species	ulcerative colitis; 5-amino salicylic acid; enteric coating; encapsulation; mucosal delivery | inflammatory bowel disease; encapsulation; mucosal delivery; drug loading and release; murine model
5	43	0.684	2021	nlrp3 inflammasome	ulcerative colitis; lipid nanoparticles; drug delivery efficiency; targeted therapy; oral administration | inflammatory bowel disease; targeted drug delivery; pathological features; inflammatory cytokines; intestinal tight junction proteins

6	19	0.817	2021	drug delivery system	inflammatory bowel disease; natural compounds; acute inflammation; nano-drug delivery; polymeric nanoparticle | ulcerative colitis; drug delivery system; zingiber officinale; oxidative stress; natural compounds
7	17	0.799	2021	polymeric nanoparticles	ulcerative colitis; nano-drug delivery; colorectal cancer; ionic gelation; drug delivery systems | inflammatory bowel disease; drug delivery; ionic gelation; drug delivery systems; risk assessment

Size: the number of articles in each cluster; Silhouette: the average contour value of clustering, it is generally believed that the clustering category with S > 0.5 is reasonable, and S > 0.7 means that the clustering is convincing; LLR: Log-likelihood ratio.

Table S6 The most trafficked keyword for the top five modules each year

Year	2000	2001	2002	2003	2004	2005	2006	2007	2008	2009
Total modules	00	-	4	6	7	6	9	9	11	9
module1	-	macrophages	gastrointestinal_ph_profiles	-	-	e_selectin_immunoconjugate	chol-but_sln	animal_models	colonic_mucosa	cyclophosphamide
module2	-	-	-	-	-	biodegradable_microparticles	gpr41_and_gpr43_receptors	drug_delivery	brain_tumours	experimental_colitis
module3	-	-	-	-	-	ulcerative_colitis	in_vitro	-	c_reactive_protein	microparticles
module4	-	-	-	-	-	-	nf_kappa_b	-	-	immune_regulating_cells
module5	-	-	-	-	-	-	phase_i	-	-	-
**Year**	2010	2011	2012	2013	2014	2015	2016	2017	2018	2019
Total modules	15	16	13	14	12					
module1	active_crohns_disease	immune_regulating_cells	inflammation	experimental_colitis	oral_drug_delivery	inflammatory_bowel_disease	polymeric_nanoparticles	nanoparticles	drug_delivery	plga_nanoparticles
module2	colon_targeting	nanoparticles	biodegradable_nanoparticles	immunogenicity	targeted_drug_delivery	drug_delivery	targeted_drug_delivery	curcumin	microspheres	ph_sensitive_nanoparticles
module3	inflamed_colonic_mucosa	oral_delivery_system	drug	chemopreventive_agent	redox_nanoparticle	ca_alginate_microparticles	drug_delivery_systems	intracellular_delivery	drug_delivery_system	drug_delivery
module4	drug_transportation	colon_specific_delivery	drug_delivery	-	nanoparticles	polymeric_nanoparticles	drug_delivery	nf_kappa_b	plga_nanoparticles	colon_targeted_delivery
module5	inflammatory_bowel_disease	dissolution_property	chain_fatty_acids	-	colon-specific_delivery	models	antioxidant	bioavailability	antioxidant	toll_like_receptors
**Year**	2020	2021	2022	2023	2024	2025	-	-	-	-

Total modules	15	16	13	14	14	-	-	-	-	-
module1	oral_drug_delivery	biodegradable_nanoparticles	microbiota	negatively_charged_liposm	intestinal_inflammation	formulation	-	-	-	-
module2	acacia_saligna	oral_nanoparticle_delivery_system	lipid_nanoparticles	hybrid_nanoparticles	colon_targeting	lipid_nanoparticles	-	-	-	-
module3	reactive_oxygen_metabolites	murine_model	drug_delivery_system	drug_delivery_system	nano particles	nlrp3_inflammasome	-	-	-	-
module4	active_targeting	mesoporous_silica_nanoparticles	cytokines	microspheres	ph_sensitive_nanoparticles	drug	-	-	-	-
module5	macrophages	nanocomposite	microspheres	antioxidant_activity	dysfunction	encapsulation	-	-	-	-

Table S7 Summary of emerging topics

ClusterID	Size	Silhouette	Average Year	Label (LLR)	Representative keywords
0	136	0.759	2021	inflammation	ulcerative colitis; yeast cell wall microparticles; co-loaded rhein; cell membranes; natural compounds | inflammatory bowel disease; gut microbiota; emerging frontiers; research trends; aromatic functional groups
2	107	0.639	2018	sulfasalazine	inflammatory bowel disease; treatment mechanism; advanced complementary therapy; colon-targeted drug delivery system; design strategy | ulcerative colitis; colorectal cancer; nano-drug delivery; cell membranes; alginate
3	98	0.759	2020	targeted drug delivery	ulcerative colitis; reactive oxygen species; biomimetic nanoplatforms; second near-infrared imaging; precise therapy | inflammatory bowel disease; gut microbiota; precise therapy; targeting therapy; natural flavonoid compounds
6	41	0.886	2019	immunoregulation	ulcerative colitis; 5-amino salicylic acid; enteric coating; metabolite analysis; biomucoadhesive nanoliposome | inflammatory bowel disease; colitis-associated colon cancer; dietary supplement; precancerous lesions; lamina propria
8	21	0.945	2021	optoacoustic imaging	ulcerative colitis; ramulus mori alkaloids; enhanced dna; active therapy; colonic epithelium | inflammatory bowel disease; systemic immunomodulation; mucosal barrier; gastrointestinal tract; epigallocatechin gallate
11	11	0.965	2021	hydrogel	inflammatory bowel disease; drug delivery; single-cell rna sequencing; mimetic nanoparticles; patchouli alcohol | ulcerative colitis; chitosan nanoparticle; oral drug delivery; ph-responsive polymers; gastrointestinal tract
23	5	0.996	2019	nanotherapy	ulcerative colitis; inflammatory markers; histopathological examination; turmeric-derived nanovesicles; peptide amphiphiles | turmeric-derived nanovesicles; macrophage polarization; gut microbiota; fresh herbs; inflammatory markers

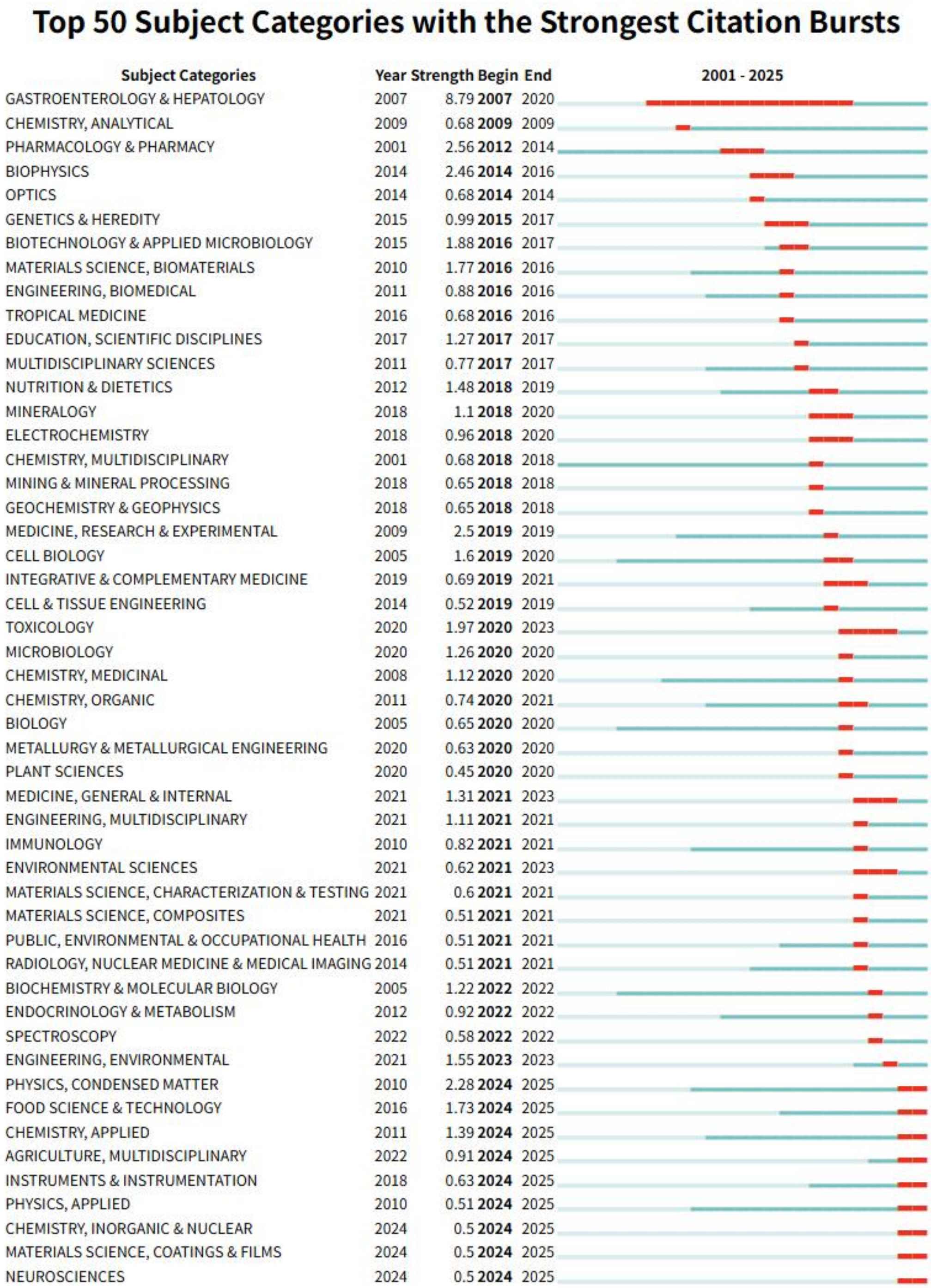

Figure S1 The top 50 most cited subject categories
